# A ventromedial visual cortical ‘Where’ stream to the human hippocampus for spatial scenes revealed with magnetoencephalography

**DOI:** 10.1038/s42003-024-06719-z

**Published:** 2024-08-25

**Authors:** Edmund T. Rolls, Xiaoqian Yan, Gustavo Deco, Yi Zhang, Veikko Jousmaki, Jianfeng Feng

**Affiliations:** 1 Oxford Centre for Computational Neuroscience, Oxford, UK; 2 Department of Computer Science, University of Warwick, Coventry, UK; 3 Institute of Science and Technology for Brain Inspired Intelligence, Fudan University, Shanghai, China; 4 Department of Information and Communication Technologies, Center for Brain and Cognition, Computational Neuroscience Group, Universitat Pompeu Fabra, Barcelona, Spain; 5 Institució Catalana de la Recerca i Estudis Avançats (ICREA), Universitat Pompeu Fabra, Passeig Lluís Companys 23, Barcelona, Spain; 6 Aalto NeuroImaging, Department of Neuroscience and Biomedical Engineering, Aalto University, Espoo, Finland

**Keywords:** Hippocampus, Spatial memory, Network models

## Abstract

The primate including the human hippocampus implicated in episodic memory and navigation represents a spatial view, very different from the place representations in rodents. To understand this system in humans, and the computations performed, the pathway for this spatial view information to reach the hippocampus was analysed in humans. Whole-brain effective connectivity was measured with magnetoencephalography between 30 visual cortical regions and 150 other cortical regions using the HCP-MMP1 atlas in 21 participants while performing a 0-back scene memory task. In a ventromedial visual stream, V1–V4 connect to the ProStriate region where the retrosplenial scene area is located. The ProStriate region has connectivity to ventromedial visual regions VMV1–3 and VVC. These ventromedial regions connect to the medial parahippocampal region PHA1–3, which, with the VMV regions, include the parahippocampal scene area. The medial parahippocampal regions have effective connectivity to the entorhinal cortex, perirhinal cortex, and hippocampus. In contrast, when viewing faces, the effective connectivity was more through a ventrolateral visual cortical stream via the fusiform face cortex to the inferior temporal visual cortex regions TE2p and TE2a. A ventromedial visual cortical ‘Where’ stream to the hippocampus for spatial scenes was supported by diffusion topography in 171 HCP participants at 7 T.

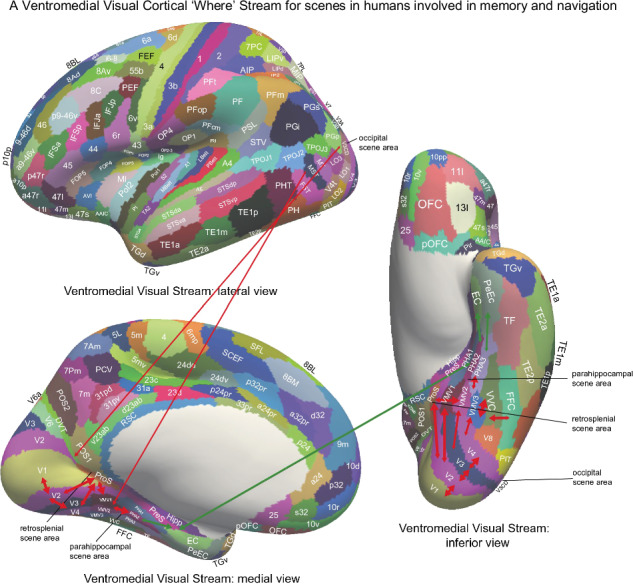

## Abstract (web summary)

A ventromedial cortical ‘Where’ visual pathway in humans for spatial scenes is revealed with MEG. It is fundamental to episodic memory and navigation.

## Introduction

**Fig. 1:**
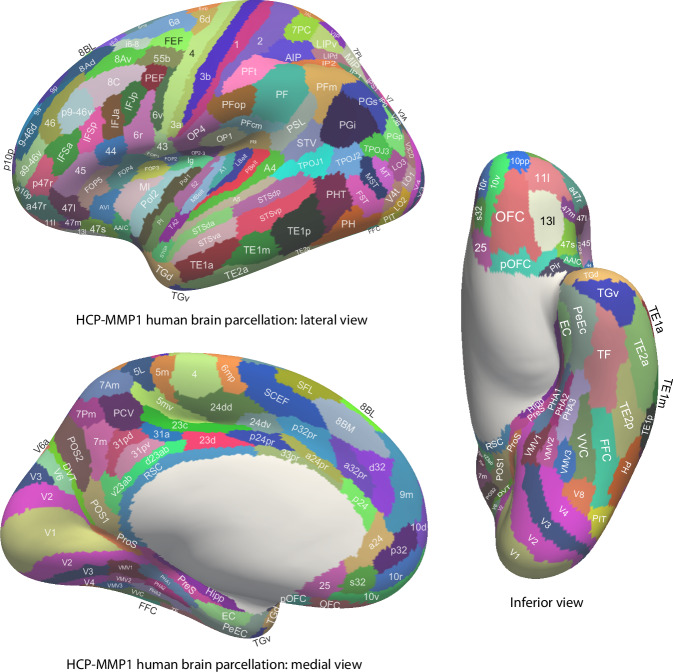
Cortical regions in the human connectome project multimodal parcellation atlas (HCP-MMP)^55^ and its extended version HCPex^120^. The cortical regions are shown on images of the human brain with the sulci expanded to show the regions within the sulci. Table S1 shows abbreviations for the cortical regions. For comparison, Fig. S1 part 5 shows the labels on the human brain without the sulci expanded. Fig. S1 parts 1–4 shows labelled coronal slices of the human brain (this figure was produced by Edmund T. Rolls and Chu-Chung Huang^63^ from data made available as part of the HCP-MMP^55^ and is available open access in ref. ^38^).

The human hippocampus (Hipp) is involved in episodic memory, our memory for past events^1,2^, and in navigation^3–5^. Much of our understanding of the Hippocampus has been based on the place cells found in rodents such as rats and mice, which encode the place where the rodent is located^3–8^. However, there is now mounting evidence in the primate including the human Hipp for neuronal spatial view representations for the location being viewed in spatial scenes^6,9–24^. Consistent with this, in human neuroimaging, a parahippocampal place area (PPA) is activated by viewed scenes, not the place where the individual is located^25–33^. Indeed, because it responds to viewed scenes, the region might better be known as the parahippocampal scene area (PSA)^6,34^, and is in ventromedial cortical regions VMV1–3 and medial parahippocampal regions PHA1–3^25,33^ (see Figs. 1 and S1).

The difference between rodents and humans is potentially very important for understanding human hippocampal systems for memory and navigation, which are very different if view-based rather than place-based computations are being used. For example, navigation in humans may be performed in part by using viewed landmarks rather than self-motion update of place^35,36^. Further, the computations involved in setting up representations of scenes are likely to involve forming feature combinations based on visual inputs that define parts of scenes, and linking these viewed parts together^6,36,37^. Because of these issues and major differences between rodents and primates including hippocampal representations^36,38,39^, it is important to understand better the cortical pathways that are involved in building scene representations in humans, which in turn has implications for what computations are performed, and how^38,40^.

In addition to the Hippocampus and the parahippocampal place (scene) area, another key brain area involved in human scene perception is the retrosplenial complex^41^ (also referred to as the medial place area^42^), which is located in regions ProStriate cortex (ProS) and dorsal visual transitional cortex (DVT) in the Human Connectome Project Multimodal Parcellation (HCP-MMP) of the cerebral cortex^25,33,34^ (see Figs. 1 and S1). There is also an occipital place area^43^ (also known as a transverse occipital sulcus region^44^) on the lateral occipital surface, which is located in or close to V3CD (including parts of IP0, V3B, V4, and LO1 in the HCP-MMP parcellation^25,34^. In addition, the caudal inferior parietal lobule is implicated in scene memory^42,45^. Functional connectivity analysis while participants were in the resting state or were looking at scenes or movies of scenes shows that these cortical regions are strongly connected with each other^6,29,33,34,42,45–50^. Further studies also show differences between posterior and anterior scene regions in connectivity, with the occipital scene area, ventromedial visual regions (VMV1–2 and VVC) and the retrosplenial scene area (ProS and DVT) having strong functional connectivity with early visual cortices such as V1–V4, while the PSA (especially PHA1–3) has strong connectivity with the Hipp^6,29,34,42,46–49,51,52^.

In this research, we present evidence on the cortical connectivity of the human Hippocampus to address these issues, and go beyond previous functional magnetic resonance imaging (fMRI)-based research on hippocampal system connectivity in humans^53^ by utilising the fast neuroimaging method magnetoencephalography (MEG) which together with a machine learning approach to measuring effective connectivity enables the directionality of the connectivity^54^ when scenes are being viewed to be measured; by utilising the Human Connectome Project Multimodal Parcellation atlas (HCP-MMP) which defines 360 cortical regions based on anatomy, functional connectivity, and task-related activations and so provides a framework for specifying which cortical regions have connectivity^55^; by presenting quantitative evidence for the connectivity between all 360 cortical regions in a new approach to describing connectivity rather than the functional connectivity measured with fMRI from a few seed regions^53^; and by complementing the MEG effective connectivity measurements with MEG functional connectivity measurements, and with diffusion tractography which uses high resolution 7-T MRI to following fibre pathways anatomically in the human brain^56^.

Previous research on the visual pathways that reach the Hippocampus has involved measuring effective, that is directed, connectivity with resting-state fMRI neuroimaging^48,57^. However, fMRI is inherently slow, with a time to measure a change in the BOLD signal to help in the calculation of effective connectivity in the order of 2 s^48,57^. For that reason, effective connectivity in the visual pathways was then measured with MEG^54^ with the data sampled at 20 ms in 88 participants in the HCP^58^. Although visual stimuli were being shown during the collection of the MEG data^58^ for that analysis^54^, the only visual stimuli used with MEG were faces and tools^58^. Because at least the functional connectivity can differ depending on which visual stimuli are being shown^33^, the new investigation described here was performed in which new MEG data were collected from 21 participants while scenes were being shown, and with 1 ms temporal resolution, for MEG data with scenes is not available from the HCP. The visual stimuli we used of scenes were in fact those used for fMRI data collection by the HCP^59^, and we have performed an analysis of these fMRI data which show activations and functional connectivity in medial temporal lobe regions in the HCP-MMP atlas^33^. In the present MEG investigation, the effective connectivity was also measured to faces, to provide a comparison of the pathways activated to those activated by scenes.

In summary, the aim of the present investigation was to trace the cortical regions through which spatial scene information reaches the Hippocampus in humans, by using the fast neuroimaging modality MEG with fast sampling at 1 ms during the presentation of spatial scene visual stimuli. New MEG data were collected, as the HCP did not use scene stimuli with MEG.

In the present investigation, the effective connectivities were measured between 30 visual cortical regions in the HCP-MMP^55^. The HCP-MMP atlas is a detailed parcellation of the human cortical regions, with its 360 regions defined using structural measures (cortical thickness and cortical myelin), functional connectivity, and task-related fMRI^55^. This parcellation is very useful for the human cerebral cortex as it utilises multimodal information^55^ with the definitions and boundaries set out in Glasser_2016_SuppNeuroanatomy.pdf^55^, and as it is being used for much new research on cortical function and connectivity, which can all be placed in the same framework^25,33,38,48,49,57,60–69^. The boundaries, tractography, functional connectivity and task-related activations of visual cortical regions with the HCP-MMP atlas are available^55,70,71^, but the effective and functional connectivity measures here are new, as they are based on presenting visual stimuli of spatial scenes and faces in a new set of participants with MEG data with sampling at 1 ms.

In the present investigation, effective connectivity was measured utilising correlations between the signals between different brain regions measured with delays, as in previous investigations^48,54,57^. A whole-brain Hopf model of the simultaneous and delayed correlations between cortical regions produces what we term a generative effective connectivity matrix, as it can generate the functional connectivities and the delayed functional connectivities^57,72,73^ as described in the Methods. It is highly relevant to this MEG investigation that the characteristic timescale for the computations performed by a cortical region is approximately 15 ms^38,74^, given that this is the timescale for the recurrent collateral connections between nearby pyramidal cells to operate for local attractor dynamics^38,75–77^, so analysis with MEG which provides data on the scale of 1–10 ms is very useful.

The results focus on the key new findings of this investigation, which are about the directionality of the effective connectivity when scenes are being viewed from V1 via the several stages of the ventromedial visual stream including the ventromedial visual regions VMV1–3 and the medial PSAs in PHA1–3 to the Hippocampus, for this has not previously been investigated with MEG. This is an important issue, for MEG is sufficiently fast, with the 1 ms acquisition used here, to follow the progression through visual cortical regions of the signal produced when scenes are shown. The use of MEG is important, for the directionality of the effective connectivity when measured with resting-state fMRI for faces, places, tools and body parts shows as the reverse of what is expected^48^, and this is probably related to the slow time course of fMRI which means that much of what is measured with fMRI resting state effective connectivity is the top–down effects from the top of the visual hierarchy where short-term memory keeps representations active^54,78^. The previous magnetoencephalography available from the HCP is available with the visual stimuli only for faces and tools^54^, and that is why we performed the investigation described here, to measure the directionality of the effective connectivity for scenes as that has not been measured before with MEG. We ran a new group of participants especially for the present investigation in which scenes were the stimuli and MEG was used, and that is thus the focus of the results described in the present investigation, and detailed statistical analyses for directionality were performed for scenes with MEG. For comparison, MEG responses to faces were included in the present study, but there is less emphasis on this in the results here, for the effective connectivity to faces has been measured previously with MEG, with HCP data^54^. We note that the localisation of signal into particular cortical regions is likely to be more accurate with fMRI than with MEG, and so rely on an fMRI study with faces, scenes, tools and body parts in 956 HCP participants for more accurate measures of the exact cortical regions in the HCP-MMP parcellation that are selectively activated by faces, scenes, tools, and body parts^33^.

## Results

### MEG Effective connectivity of visual cortical regions when viewing spatial scenes vs faces

#### Mean effective connectivity when viewing spatial scenes

**Fig. 2:**
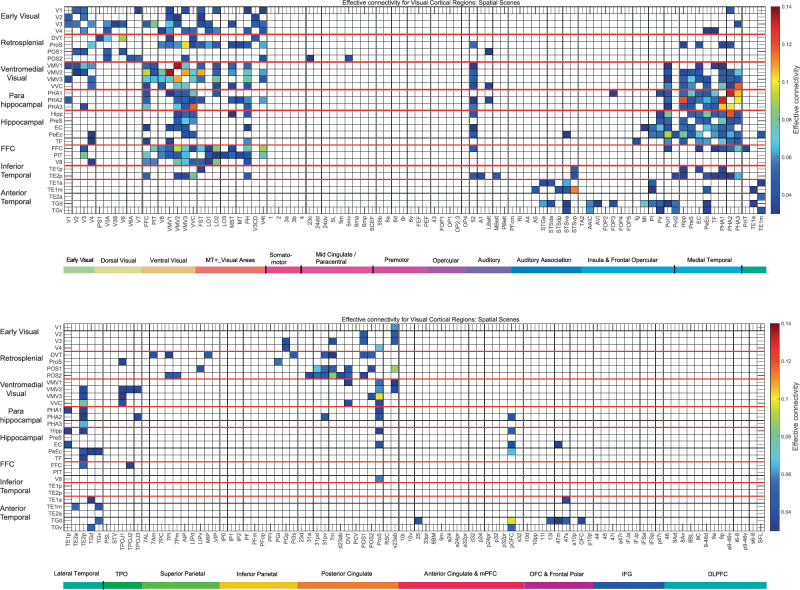
MEG Effective connectivity for visual cortical regions (the rows) with 180 cortical regions (the columns) in both hemispheres when viewing spatial scenes. The effective connectivities are the mean across both directions for every pair of cortical regions. Effective connectivities of <0.03 are shown as white to help reveal the main effective connectivities between the cortical regions. The effective connectivity map is scaled with 0.14 as the maximum. The effective connectivity in the top panel is for the first set of 90 cortical regions; and in the lower panel for the second set of 90 cortical regions. The abbreviations for cortical regions are shown in Table S1. Horizontal red lines separate the groups of visual cortex regions. Group 1: (top) early visual cortical areas V1–V4 in the HCP-MMP atlas; Group 2: cortical regions in the retrosplenial complex; Group 3: ventromedial visual cortical regions; Group 4: parahippocampal cortex regions; Group 5: hippocampal and related regions; Group 6: intermediate ventrolateral cortical visual regions FFC (fusiform face cortex), PIT (posterior inferior temporal cortex), and V8. Group 7: inferior temporal visual cortex regions TE2p and TE1p. Group 8: anterior temporal lobe multimodal regions including the temporal pole TGd and TGv. The coloured labelled bars indicate the cortical divisions in the HCP-MMP atlas^55^. The order of the cortical regions on the horizontal axes is that in Huang, ref. ^120^.

**Fig. 3:**
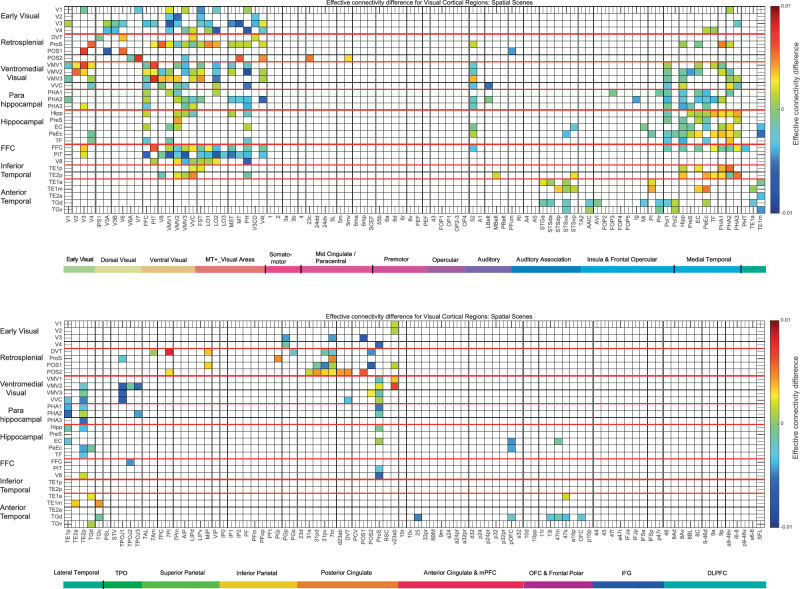
Difference in the directionality of the effective connectivity for visual cortical regions (the rows) with 180 cortical regions (the columns) in both hemispheres when viewing spatial scenes. For a given link, the effective connectivity difference is shown as positive when the connectivity is stronger in the direction from column to row. For a link, the effective connectivity difference is shown as negative when the connectivity is weaker in the direction from column to row. The threshold value for any effective connectivity difference to be included is 0.0005 for the connectivities shown in Fig. 2. This threshold was chosen to help show which differences were greater than or lesser than zero. Table S1 shows the abbreviations for the cortical regions, and the cortical regions are shown in Figs. 1 and S1. The effective connectivity difference in the top panel is for the first set of 90 cortical regions; and in the lower panel for the second set of 90 cortical regions. The conventions are as in Fig. 2.

The effective connectivity of 30 visual cortical regions in the HCP-MMP atlas when viewing spatial scenes with all cortical regions is shown in Fig. 2, and for comparison when viewing faces in Fig. S2. The visual cortical regions are grouped for convenience as shown in Fig. 2 and as described in the Methods. The effective connectivities shown in Fig. 2 are the mean for both directions, i.e. the mean of the column-to-row effective connectivity and of the row-to-column effective connectivity. The differences in the connectivity in the two directions are then shown in Fig. 3, where yellow/red/brown colour indicates greater effective connectivity from column to row. Figure 2 shows that when visual scenes are being viewed in the 0-back memory task, in the early visual cortical regions, there is effective connectivity of V1 with V2, V3, VMV1, VMV2, and PHA2. V2 has effective connectivity with V3, POS1, VMV1, and VMV2. V3 has effective connectivity with V4, POS1, VMV1, VMV2, PHA3, FFC, and PIT. V4 has effective connectivity with ProS, VMV1, VMV3, VVC, PeEc (perirhinal cortex), TF (lateral parahippocampal), and V8.

Figure 2 also shows that when visual scenes are being viewed, for the Retrosplenial Regions, ProS has effective connectivity with V4, VMV1–3, and VVC, linking ProS to ventromedial visual cortical stream processing. Interestingly, ProS also has some effective connectivity with MT+ complex regions with visual motion sensitivity, especially LO1 and V4t. ProS also has effective connectivity with the medial parahippocampal cortex PHA1 and PHA2, the entorhinal cortex (EC), and the Hipp. In contrast, the DVT region has effective connectivity with Dorsal Stream Regions IPSI, V3A, and V6; and with superior parietal 7Pm, 7Pl, and MIP, suggesting that DVT is involved in visual motion analysis. POS1, which is close to ProS, and which can be activated by scenes^33^, has effective connectivity with V2 and V3, with DVT and v23ab, and with V3A and V6. v23ab, which is in the same general retrosplenial region, does have interesting effective connectivity not only with POS1, but also with V1, V2, V3, and ventromedial visual regions VMV1 and VMV2 (Fig. 2).

The ventromedial visual regions VMV1–3 and VVC have effective connectivity with V1, V2, and more with V3 and V4, and with each other (Fig. 2). The ventromedial visual regions also have effective connectivity with medial parahippocampal regions PHA1–3, and further with the PeEc, EC, and Hipp, when viewing spatial scenes (Fig. 2). The ventromedial visual regions thus are in a route from early visual regions to parahippocampal and hippocampal regions when viewing spatial scenes. There is also effective connectivity with FFC, V8, PIT, and TE2p (Fig. 2).

Although fMRI may allow more accurate localization of activations and connectivities than MEG, these findings with MEG are in fact well supported by the findings with activations and functional connectivities selective for scenes when measured with fMRI^33^.

#### Mean effective connectivity when viewing faces

**Fig. 4:**
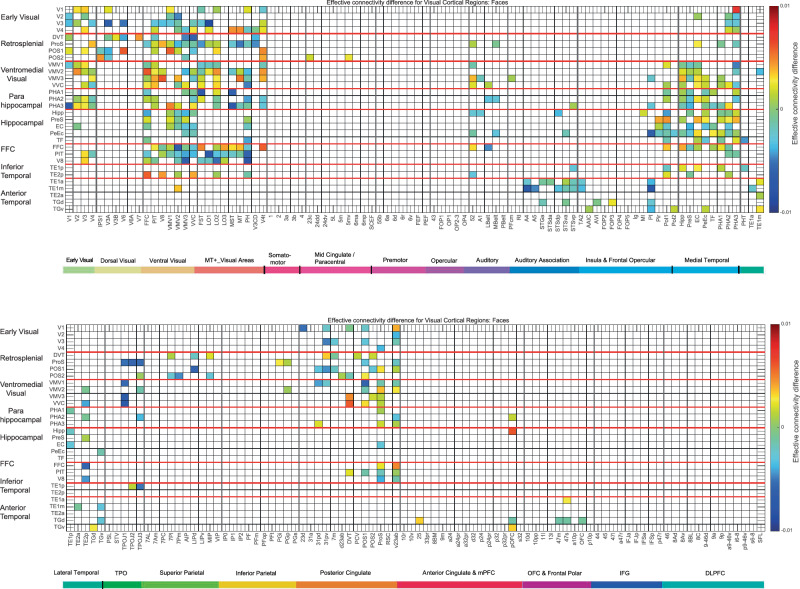
Difference in the directionality of the effective connectivity for visual cortical regions (the rows) with 180 cortical regions (the columns) in both hemispheres when viewing faces. For a given link, the effective connectivity difference is shown as positive when the connectivity is stronger in the direction from column to row. For a link, the effective connectivity difference is shown as negative when the connectivity is weaker in the direction from column to row. The threshold value for any effective connectivity difference to be included is 0.0005 for the connectivities shown in Fig. 2. Table S1 shows the abbreviations for the cortical regions, and the cortical regions are shown in Figs. 1 and S1. The effective connectivity difference in the top panel is for the first set of 90 cortical regions; and in the lower panel for the second set of 90 cortical regions. The conventions are as in Fig. 2.

The effective connectivity of 30 visual cortical regions in the HCP-MMP atlas when viewing faces is shown in Fig. S2. Overall, these mean effective connectivities when viewing faces (Fig. S2) are rather similar to those when viewing spatial scenes (Fig. 2), and it is when the differences in the directions of effective connectivity between every pair of cortical regions are considered that the effective connectivities for spatial scenes and faces are found to be different (see next section, and Figs. 3 and 4). However, comparison of Figs. 2 and S2 does provide some indication that when viewing faces there is higher effective connectivity in inferior temporal cortex TE1p and TE1a, and the anterior temporal and temporal pole regions than when viewing scenes.

These MEG results with faces are provided just for comparison with the results with scenes. For accurate localization of activations and functional connectivities that are selective for faces vs scenes, this is better provided by the fMRI analyses in 956 HCP participants^33^.

#### The directionality of the effective connectivities when viewing spatial scenes (Fig. 3) compared to faces (Fig. 4)

The directionalities of the effective connectivities of 30 visual cortical regions in the HCP-MMP atlas when viewing spatial scenes are shown in Fig. 3, with yellow/red/brown indicating higher effective connectivity from a column to a row than vice versa. The directionality differences from early visual regions such as V2, V3, and V4 to retrosplenial visual regions such as ProS and POS1, and to ventromedial visual regions VMV1–3 and VVC, are higher during viewing of scenes (Fig. 3) than faces (Fig. 4). Further, the directionality differences from the parahippocampal cortex regions PHA1–3 to hippocampal regions including the Hipp, EC, and PeEc are higher during viewing of scenes (Fig. 3) than faces (Fig. 4). In addition, the directionality differences from posterior cingulate regions implicated in memory such as 31pd, 32pv, d23ab, and v23ab to retrosplenial regions such as ProS, POS1, and POS2 are higher during viewing of scenes (Fig. 3) than faces (Fig. 4).

The most important statistical comparisons are for the directionality of effective connectivity between cortical regions in the ventromedial visual cortical stream when scenes are being viewed, as that is a key aim of this paper using MEG, and to support what is shown in Fig. 3. The statistical comparisons were based on two-tailed paired *t*-tests performed across the 21 participants of whether there was higher effective connectivity in one direction than another between sets of cortical regions in preplanned comparisons. It was found that there was stronger effective connectivity from V3-V4 to the ProS, the key region in the retrosplenial scene area, than in the backward direction (*t* = 3.51, *p* = 0.0022, d*f* = 20). There was stronger effective connectivity from V3–V4 to the ventromedial visual regions VMV1, VMV2, VMV3, and VVC than in the backward direction (for example, V3–V4 to VMV1 *t* = 2.62, *p* = 0.016, d*f* = 20). There was stronger effective connectivity from V3 to the parahippocampal visual regions PHA1, PHA2 and PHA3 than in the backward direction (*t* = 4.09, *p* = 0.0006, d*f* = 20). There was also stronger effective connectivity when viewing scenes from the PSA regions PHA1-PHA3 to the Hipp than in the backward direction (*t* = 2.97, *p* = 0.008, d*f*=20). Thus this MEG investigation provided new evidence that when viewing scenes, the effective connectivity is from early visual cortical regions such as V2–V4 to a retrosplenial region the ProS; to ventromedial visual cortical regions VMV1, VMV2, VMV3, and VVC; and to medial parahippocampal cortex regions PHA1, PHA2, and PHA3. Interestingly, it was also possible to show onward effective connectivity from the medial parahippocampal regions PHA1–3 to the Hipp when scenes were being viewed in the 0-back memory task. The directionality was made evident by using a delay of tau = 20 ms when measuring the effective connectivity, and this is in the order of time that it takes for visual information to cross one or two stages in a visual processing hierarchy^38,54,75,79^. With tau = 10 ms, the directionality was similar, but the effect size for the directionality was smaller, as expected.

Similarly, when viewing faces (Fig. 4), there was stronger effective connectivity in the ventrolateral ‘What’ (face and object) pathway in the direction from early visual cortical regions (e.g. V3) to PIT (posterior inferior temporal) and V8; and from PIT, V8 and fusiform face cortex (FFC) to TE2p, which is at the highest level in the inferior temporal visual cortex where the processing is mainly unimodal, as well as to the more anterior multimodal semantic regions such as TE2a^54^. Some key statistical comparisons are as follows: The effective connectivity from FFC, PIT and V8 to TE2p is stronger in that direction than vice versa (*t* = 2.19, *p* = 0.04, d*f* = 20). The effective connectivity from FFC, PIT and V8 to TE2a is stronger in that direction than vice versa (*t* = 6.85 *p* = 10^−5^, d*f* = 20).

The new evidence from magnetoencephalography is thus consistent with the directionality of the flow of information based on differences in effective connectivity in a ventromedial visual cortical stream from early visual cortical regions (V2–V4), to retrosplenial regions such as ProS and POS1, to ventromedial visual cortical regions VMV1–3 and VVC, and via parahippocampal regions PHA1–3, which in turn have connectivity to the Hippocampus and related regions such as the entorhinal and PeEc (Fig. 3).

Further, when viewing faces, the flow of information is from early visual cortical regions (and interestingly from visual motion regions in the MT+ complex even though the faces were stationary) to the regions close to the level of FFC such as PIT and V8, and from the FFC, PIT, and V8 to inferior temporal cortex TE2p and anterior temporal cortex TE2a (Fig. 4).

#### The functional connectivities when viewing spatial scenes (Fig. S3)

For completeness, the MEG functional connectivities of the visual cortical regions are shown when viewing spatial scenes in Fig. S3. The threshold has been set so that the proportion of connectivities shown is the same as for the effective connectivities in Fig. 2, 0.087, to facilitate comparison between the two. The functional connectivity matrix (Fig. S3) is rather similar to the mean effective connectivity matrix (Fig. 2), which also does not capture the directionality of the connectivity. What is quite interesting about the similarity is that this may imply that a functional connectivity matrix does reflect considerably the effective connectivity matrix, and that might have implications for the interpretation of what a functional connectivity matrix can show. However, it must be remembered that in the Hopf effective connectivity algorithm, the effective connectivity matrix is being optimized that will best generate the functional connectivity matrix and the functional connectivity matrix delayed by tau = 20 ms, so the similarity is not surprising. A real difference though that makes the effective connectivity matrix useful is that it generates the set of connection strengths that can best generate the functional connectivity matrices without and with a delay, setting the other connectivities to zero, and thereby the mean effective connectivity matrix allows a threshold to be set in the functional connectivity matrix about what may be relevant, and this is useful for the functional connectivity matrix has continuous values in the range −1 to +1, and it is otherwise difficult to know what range of values may be useful.

#### The tractography of the visual cortical regions

**Fig. 5:**
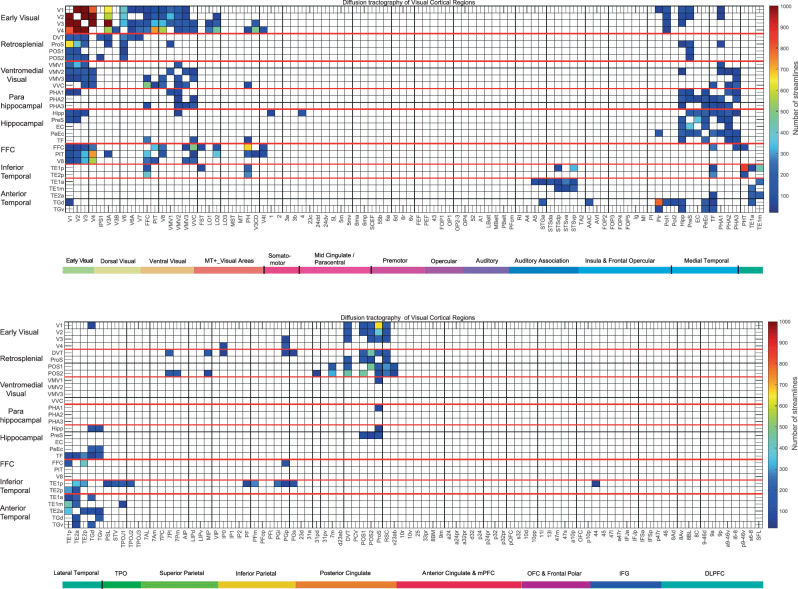
Connections between the visual cortical regions (the rows) with 180 cortical regions (the columns) in both hemispheres are shown by diffusion tractography. The layout is the same as in Figs. 2–4. The number of streamlines shown was thresholded at 50; values less than this are shown as white to reveal the main connections. The colour bar was thresholded at 1000 streamlines. Table S1 shows the abbreviations. The conventions are as in Fig. 2.

The diffusion tractography matrix for these visual cortical regions is shown in Fig. 5, and may be useful in providing evidence about which of the effective connectivities in Figs. 2–4 may be mediated by direct vs trans-synaptic connections. The tractography provides evidence for direct connections from V1, V2, and V3 to ProS and most of the other retrosplenial regions in Fig. 5. There is also evidence for some direct connection between V1 and V4 and the ventromedial visual cortical regions, but much less with parahippocampal regions PHA1–3. ProS is shown as having connections with VMV1. The ventromedial visual cortical regions VMV1–3 and VVC have connections with the parahippocampal regions PHA1–3. The medial parahippocampal regions PHA1–3 have connections with the Hipp, and to a lesser extent with the PeEc and EC. These connections are consistent with what has been described by the effective connectivities shown in Figs. 2 and 3 in providing evidence for a staged hierarchically organised ventromedial cortical visual stream involving connectivity from early visual cortical regions V1–V4, to retrosplenial regions including ProS, to ventromedial visual cortical regions VMV1–3 and VVC, to parahippocampal regions PHA1–3, to the Hipp. Moreover, this ventromedial visual cortical pathway is implicated in spatial scene processing in that its directed effective connectivity is greater when viewing scenes than faces (Figs. 2 and 3); by the activations of retrosplenial regions ProS and POS1 when scenes are viewed^25,33^ in what is termed the retrosplenial scene area; by the activations of ventromedial visual cortical regions VMV1–3 and VVC, and of parahippocampal regions PHA1–3, when spatial scenes are viewed in what is termed the parahippocampal place or scene area^25,33^; and by the presence of spatial view cells in the macaque parahippocampal gyrus (as well as Hipp)^6,9–12^.

The tractography also shows in a ventrolateral visual cortical stream (Fig. 5) connections between V4 and V3 (and to a smaller extent V2 and V1) and FFC, V8 and PIT; with FFC having connections with inferior temporal visual cortex TE1p and TE2p; which in turn have connections with anterior temporal lobe regions including TE1a, TE1m, and TE2a. These anterior temporal lobe regions also have connections with regions in the STS (STSGa to STSvp). The ventrolateral pathway via FFC to the inferior temporal cortex is implicated in face processing, partly by the evidence of greater directional effective connectivity to faces (Fig. 3) but by a wealth of other evidence^34^. The visual stream to the STS is also implicated inter alia in responses to facial expressions and to moving heads that make or break social interactions^34,80–83^.

#### Laterality differences in the functional connectivity of the visual cortical regions

**Fig. 6:**
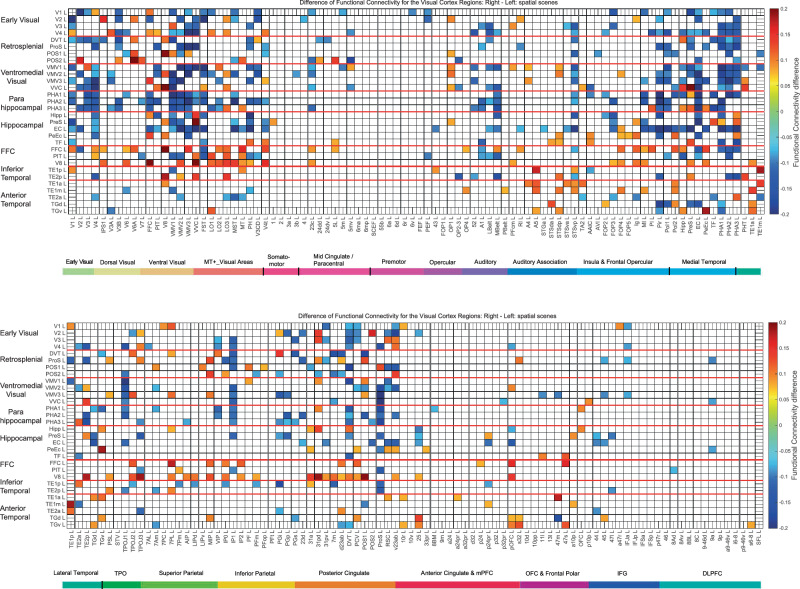
Difference of the MEG functional connectivity for the Right–the Left hemispheres for visual cortical regions (the rows) with 180 cortical regions (the columns) when viewing spatial scenes. Differences in functional connectivity of less than 0.075 are shown as blank in order to reveal the main differences.

Laterality differences in the functional connectivity when viewing scenes might provide further evidence on the specialization of different visual cortical processing streams, and are shown in Fig. 6. In these participants (who were Chinese), the functional connectivity of the ventromedial cortical regions VMV1–3 and VVC with ProS, with parahippocampal region PHA1–3, and with the Hippocampus and EC was stronger on the left. This is not inconsistent with earlier results on laterality when places/scenes are viewed, which show less lateralisation for scenes than for faces and that the PSA can have higher functional connectivity in the left hemisphere in 956 HCP participants with fMRI^33^, and this supports the current MEG analyses.

In contrast, the functional connectivity of the FFC, V8, and PIT, parts of the ventrolateral cortical visual stream involved in face and object processing^33,34,48,54^, had higher functional connectivity on the right, as does the STS system involved in face expression and movement^34,81–83^ (Fig. 6). The consistency with earlier results with fMRI on face laterality^33,84,85^ supports the current MEG analyses.

This evidence for a dissociation of pathways based on laterality supports the hypothesis that there is a ventromedial cortical visual stream for scene information to reach via ProS, the ventromedial visual cortical regions VMV1–3 and VVC, the medial parahippocampal cortex PHA1–3, and Hipp that is distinct from a ventrolateral pathway via FFC, V8, and PIT for information about faces and objects to reach the inferior temporal visual cortex in TE1p and TE1p, and more anterior temporal lobe regions including TE2a.

## Discussion

**Fig. 7:**
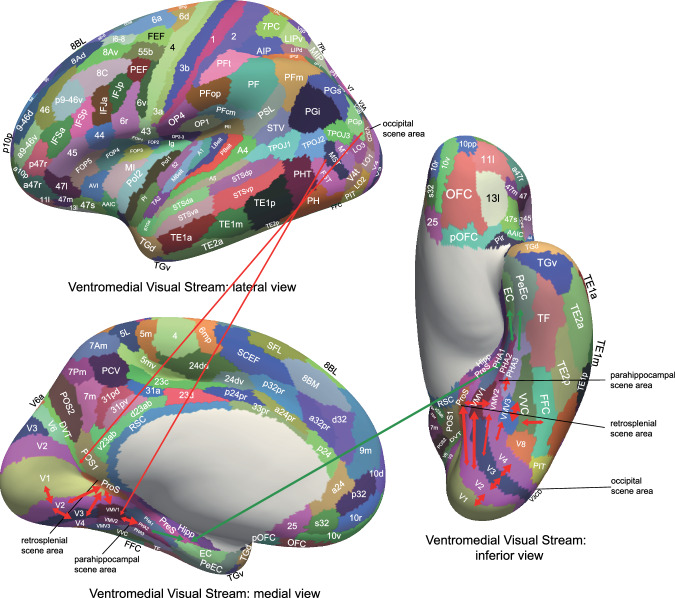
Hierarchical organisation of the ventromedial visual cortical stream measured with MEG effective connectivity when viewing spatial scenes: an overview. At a first level, after V1, V2–V4 have connectivity to the ProS and POS1 which are where in humans the retrosplenial scene area is located. At a second level, ProS has connectivity to the ventromedial visual cortical regions (VMV1–3 and VVC). These ventromedial visual cortical regions also receive effective connectivity from MT and MST etc in the dorsal visual cortical stream. At a third level, the ventromedial visual cortical regions have effective connectivity to the medial parahippocampal cortex regions PHA1–3. The medial parahippocampal cortex regions PHA1–3 also have effective connectivity from the ventrolateral visual cortical stream region FFC. The PSA is located at the intersection of the ventromedial visual regions (VMV1–3 and VVC) and medial parahippocampal regions PHA1–3. At a fourth level, the medial parahippocampal regions PHA1–3 have effective connectivity to the hippocampal memory system (arrow in green). The line widths of the arrowhead's size indicate the magnitude and direction of the effective connectivity (this figure was produced by Edmund T. Rolls using the cortical region template shown in Fig. 1).

This research used MEG during the presentation of images of spatial scenes and showed (see Fig. 7) with the HCP-MMP atlas that MEG effective connectivity reveals a ventromedial cortical visual stream from V1–V4 to the ProS where the retrosplenial scene area is located; then to the ventromedial visual cortical regions VMV1–3 and VMV; then to the parahippocampal cortex PHA1–3; and then to the Hippocampus. The parahippocampal place or scene area is located in the VMV and PHA regions^25,33^. This ventromedial cortical visual stream was supported by analysis of diffusion tractography in 171 HCP participants. For comparison, when faces were being viewed, the effective connectivity was directed from V1–V4 more to the FFC, and then to the inferior temporal cortex regions TE2p and TE2a, in a ventrolateral visual cortical stream.

These results provide an important addition to previous research^48,54^ for the following reasons. First, the direction of the effective connectivity is easier to establish with MEG with its fast time resolution of 1–20 ms than with fMRI, perhaps because of the long time delays in the order of 2 s inherent with the development of the BOLD fMRI signal which may provide time for the signal to propagate upwards, and then back down again to earlier regions in a hierarchy^54,78^. Thus although fMRI can give useful results for effective connectivity, care is needed in interpretation of the direction of the effective connectivity measured with fMRI^54^. Second, previous fMRI analyses of effective connectivity with the HCP-MMP atlas were with the resting state^48,57^, whereas the present analysis measured effective connectivity while spatial scenes were being viewed and remembered. Third, a previous MEG investigation that used MEG data provided by the HCP was for face and tool visual stimuli^54^, whereas here we acquired new MEG data in which the visual stimuli were spatial scenes. This is important, for which pairs of regions show directed effective connectivity is different for spatial scenes vs faces (see Figs. 3 and 4).

Some interesting points arise from the findings with MEG described here.

First, the ProS appears to be a key region in the ventromedial visual cortical stream for spatial scenes, because it has effective connectivity from V3 and V4, to the ventromedial cortical visual areas VMV1–3 and VVC, and with parahippocampal regions PHA1–2 (Fig. 2). In contrast, the dorsal transitional visual region DVT has less of this connectivity, and instead has effective connectivity with some dorsal visual division regions IPS1, V3A, V6, and MT+ division region PH (Fig. 2). This is an indication that DVT is more concerned with visual motion analysis than with spatial scene analysis.

Second, POS1 and v23ab are both in the general retrosplenial area, when viewing scenes have strong effective connectivity with each other and v23ab has effective connectivity with ventromedial visual cortical regions (Fig. 2), and POS1 is activated in the HCP fMRI task-related data when viewing spatial scenes^33^. This provides an indication that there may be quite an extended part of the retrosplenial area including ProS, and POS1, and perhaps v23ab given its connectivity, that is related to spatial scene processing.

Third, the differences in effective connectivity in the two directions between any pair of regions may be smaller with MEG than with fMRI. The magnitude that is measured with either depends on the tau value used for the time delay between the two FC correlations used to calculate the effective connectivity: if tau is short, then there is less time for the time-delayed connectivity to become different. Given that a tau of 20 ms was used for the MEG analyses used here and in a previous investigation^54^, and of 2 s for the fMRI analyses of effective connectivity^48^, the small difference for the two directions with MEG is expected.

Fourth, we did not attempt to measure the magnitude of the activations of different cortical regions with MEG, because the magnitude of the MEG signals can depend on the orientation of a cortical region with respect to the sensors. Instead, we rely on activations measured with fMRI to allow the activations of different cortical regions to be compared more accurately to stimuli such as faces, scenes, tools, and body parts e.g. ref. ^33^.

Fifth, the interestingly different functional connectivities shown in Fig. 6 with higher values for faces in the right hemisphere and scenes in the left hemisphere were found with Chinese participants, and these particular laterality differences might not be found in all populations, relating to differences in the organisation of language systems in the cortex in different populations. The laterality differences shown in Fig. 6 though do provide useful extra evidence that a ventromedial visual cortical pathway activated by scenes is different from a ventrolateral cortical visual pathway activated by faces etc.

It is useful to compare the approach taken here and in related recent studies on connectivity of the human medial temporal lobe using the HCP-MMP atlas^33,34,48,49,54,57,64–66,68,86^ with another recent investigation which used resting state individualized fMRI to measure functional connectivity from 3 seed regions in four participants, and described the results in terms of, for example, “the human parahippocampal area TH is preferably associated with the retrosplenial cortex rather than with the posterior cingulate cortex”^53^ (What was described as TH probably corresponds to PHA1–3 here.) In contrast, here we measured functional and effective connectivity between 30 visual cortical and medial temporal lobe regions with each other and with 150 other cortical regions in 21 participants who were viewing spatial scenes or faces for there is clear evidence with fMRI in 956 HCP participants that this affects the functional and effective connectivity and reveals different pathways^33^; expressed the results in terms of connectivity matrices using the HCP-MMP atlas which provides a foundational basis for defining the cortical regions involved and by being surface-based helps to resolve problems with the measurement of activation peaks in volume space when defined cortical regions may be in different locations with respect to sulci in different individuals; and complemented these effective and functional connectivity analyses with diffusion tractography performed in the same HCP-MMP space with high resolution 7-T MRI. We suggest that the use of connection matrices to provide quantitative values for the connectivity between large numbers of well-defined cortical regions as used here and elsewhere^33,34,38,48,49,54,56,57,64–69,87^ may provide an important development in the way that anatomical/connectivity evidence can be presented in future, with part of its attractiveness that cortical activations etc can be shown in the same well-defined cortical space, allowing comparison between different studies and obtained with different methods.

A key conceptual point made by the findings described here is that they provide support for the hypothesis that in humans (and other primates) a key spatial pathway for ‘Where’ information to reach the hippocampal memory and navigation system is involved in a ventral stream type of processing in which images on the retina are analysed in terms of spatial visual features being looked at in the world^36^. The coding is for information about spatial scenes, as shown by fMRI for the retrosplenial and PSAs in humans that are activated by what the human looks at, not the place where the individual is located^25–33^. Moreover, the spatial view information in the parahippocampal gyrus and Hippocampus is about the location in the scene, as shown by neuronal recordings in non-human primates from spatial view cells^6,9–18,24,39,88–90^. The representation of the location in a scene being looked at encoded by these spatial view neurons provides a ‘Where’ representation for hippocampal episodic memory in primates including humans, which typically involves associations between what face or object is being looked at, and where it is in a spatial scene^6,13,88–91^. In great contrast, in rodents, the place where the individual is located is represented in the Hipp, and update may be by path integration over places based on body movements^5–8,92^. This underlines the great importance of analysing the ‘Where’ cortical pathways in primates including humans as in the present paper, for what is found in rodents may be considerably different^36,38^.

This ventromedial cortical ‘Where’ visual pathway for scenes is hierarchically organised as shown by the following. First, the connectivity is hierarchical with for example early visual cortical regions V1–V4 having prominent effective connectivity with VMV regions; then VMV regions having prominent effective connectivity with PHA regions; and then PHA regions having prominent effective connectivity with the Hippocampus (Fig. 3). Second, the effective connectivity has directionality that is generally in this forward direction through these stages from early visual cortical regions to the Hippocampus (Fig. 3). Third, as noted above, the early visual cortical regions have relatively small receptive fields that are in retinotopic coordinates and that respond to stimuli such as bars and curves^93,94^, whereas by the medial parahippocampal gyrus (PHA1–3 in humans) and the Hippocampus spatial view neurons have allocentric representations to locations being viewed in scenes^6,9–12,39,95^, and in the Hippocampus can combine this with object and reward information to encode episodic memories^36,40,89,90,96,97^.

The evidence that the ventromedial cortical visual stream for scenes leading to the medial parahippocampal cortex (PHA1–3) is distinct from the ventrolateral cortical visual stream for faces and objects leading to the lateral parahippocampal cortex (TF) and the anterior temporal lobe semantic systems includes the following. First, the effective connectivities for VMV and PHA regions and PHA connectivity to the Hippocampus tend to be higher for scenes (Fig. 2) than for faces (Fig. S2), whereas the effective connectivities for faces tend to be higher than for scenes in FFC, V8, and PIT cortical regions (Figs. 2 and S2). This particular point is greatly strengthened by the analysis of scene-selective and face-selective activations and functional connectivities when measured with fMRI in a much larger group of 956 participants^33^. Second, the identification of the scene pathway as a separate visual pathway is confirmed by cluster and multidimensional scaling analyses based on resting-state functional connectivity^98^. Third, we have developed a dynamical graph approach to the analysis of cortical streams that enables whole networks rather than pairwise effective connectivities to be analysed, and this provides evidence from our effective connectivity investigations with 171 HCP participants imaged at 7 T^48,49,57^ and from 956 HCP participants performing a working memory task with scenes, faces, tools or body parts as the stimuli for separate pathways and indeed whole networks to the Hippocampus for scenes (via VMV and PHA regions), and for faces via the FFC and TF regions^78^. Fourth, in the laterality section of the Results of this paper, the functional connectivity of the ventromedial cortical regions VMV1–3 and VVC with ProS, with parahippocampal region PHA1–3, and with the Hippocampus and EC was stronger on the left (Fig. 6). In contrast, the functional connectivity of the FFC, V8, and PIT, parts of the ventrolateral cortical visual stream involved in face and object processing, had higher functional connectivity on the right (Fig. 6). This evidence for a dissociation of pathways based on laterality supports the hypothesis that there is a ventromedial cortical visual stream for scene information to reach via ProS, the ventromedial visual cortical regions VMV1–3 and VVC, the parahippocampal cortex, and Hippocampus, that is distinct from a ventrolateral pathway via FFC, V8, and PIT for information about faces and objects to reach the inferior temporal visual cortex in TE1p and TE1p, and more anterior temporal lobe regions. Fifth, this ‘Where’ ventromedial cortical visual stream is more activated when performing episodic memory tasks with scenes than with word-pairs^91^.

This ventromedial pathway for spatial scenes has important implications for what is computed for ‘Where’ representations in the parahippocampal cortex and Hippocampus of primates including humans. The implication is that the spatial view representations are built by combinations of spatial features that are nearby in a scene, that is which fall within the primate fovea^6,99,100^. It is proposed that then these feature combination neurons are locked together in the correct spatial arrangement in a continuous attractor network built because nearby locations in a scene will have co-firing neurons that support associative synaptic modification^36,37,101^. This raises the interesting issue of how the spatial representations change up through the ventromedial visual cortical stream for spatial scene representations. In V1–V4, the visual receptive fields are relatively small, a few degrees across, and encode simple feature combinations such as lines and edges and in V4 combinations of these may form curves^93,94,102–105^, and are retinotopic. In contrast, in the macaque parahippocampal cortex and Hippocampus, spatial view cells have larger receptive fields that may subtend many degrees of visual angle, respond to a location in a world-based scene, are relatively invariant with respect to eye position and head direction, and even of the place at which the individual is located provided of course that the spatial view field can be viewed, and can thus be described as allocentric, world-based, and not egocentric^6,9–13,106^. The implication is that along the primate (including human) ventromedial visual stream, in regions such as the retrosplenial regions and ventromedial visual cortical regions, the representations will gradually become larger, less retinotopic and more head or even eye-direction-based in world coordinates, and later more independent of the place where the individual is located provided of course that the view field in the scene can be seen. Indeed, with wide-field retinotopic mapping, the ProS, where the retrosplenial scene area is located^25,33^, is found to have a complete representation of the visual field, different from adjacent area peripheral V1 and dorsal V2^107,108^, exhibits large receptive fields that can extend 30–50° in diameter^109^, respond to very fast visual motion^110^, and is associated with peripheral scene monitoring^42,107,108,111^. The mechanisms by which these transformations may be learned include gain modulation of neuronal responses by for example eye position to convert from retinotopic to head-based coordinates, facilitated by slow learning^95,101^, and then even beyond to world eye-direction-based coordinates^112^. The proposed mechanisms are analogous to learning of invariant object and face representations in the ventrolateral visual cortical stream via the FFC to the inferior temporal visual cortex^36,38,101,113^.

The implications of this ventromedial visual cortical ‘Where’ stream for spatial scenes for understanding hippocampal function in memory and navigation in humans and other primates are developed further elsewhere^6,13,33,34,36,38,96^.

## Methods

### The visual stimuli and task

The scene and face visual stimuli were those provided by the HCP that were used in their fMRI data collection^59^, and the 0-back task used by the HCP in their MEG data collection for faces and tools^58^ was implemented (details are available at https://www.humanconnectome.org/hcp-protocols-ya-task-fmri and https://db.humanconnectome.org/app/action/ChooseDownloadResources?project=HCP_Resources&resource=Scripts&filePath=HCP_TFMRI_scripts.zip.) A 0-back task was used to ensure that the participants looked at and processed the visual stimuli by remembering the cue stimulus and indicating whether it appeared in the next ten trials of a run. Within each task block or run, first, a cue image was presented for 2.5 s to indicate the 0-back cue stimulus, and then ten trials were run for a given stimulus type, with each stimulus shown for 2.0 s followed by an interstimulus interval of 0.5 s in which the screen was blank. The ten stimuli in each block thus lasted for 25 s. During the experiment, participants received 8 blocks of scenes and eight blocks of faces in random order, with no repetition of any image. Examples of the scene and face stimuli provided by the HCP and used here are available^33^.

Twenty-four participants from Fudan University aged 19–30 years (13 females) participated in the experiment. Three of them had to be excluded because of MEG artefacts in the data, leaving 21 datasets for the final analysis. All participants were reported to be right-handed and have normal or corrected-to-normal vision. They reported to have no history of neurological disorders. The study received ethical approval from the Ethics Committee of the Institute of Science and Technology for Brain-Inspired Intelligence at Fudan University (reference number AF/SC-115/20230822). All participants provided informed written consent. All ethical regulations relevant to human research participants were followed.

### MEG data acquisition and preprocessing

MEG data were acquired on a TRIUX neo (MEGIN Oy, Finland) system at Zhangjiang Imaging Center (ZIC), containing 306 MEG sensors (102 magnetometers and 204 gradiometers). The sampling rate during data acquisition was 1000 Hz and an online band pass filter 0.03–330 Hz was applied. Prior to data recording, five Head Position Indicator coils that were attached to the forehead of the subjects, as well as three anatomical fiducial points (two preauricular points and one nasion) were digitized using the FASTRAK Digitizer system for later co-registration with MRI data.

High-resolution structural T1-weighted MRI images were acquired in a 3-T Siemens Prisma scanner at ZIC with a 3D T1 MPRAGE sequence, field of view = 192 × 240 × 256 mm, 1 mm isotropic resolution, repetition time = 2.5 s, echo time = 2.15 ms, and flip angle = 8°.

The MEG preprocessing was performed with the MNE-Python version 1.5.1 software package (https://zenodo.org/records/8322569)^114^. First, the environmental noise, recorded every day in the morning before testing, was suppressed from the raw MEG data with the spatio-temporal signal-space separation (SSP) method that is implemented in MNE-Python. A notch filter at 50 Hz and 100 Hz was then applied, followed by a band-pass filter between 1 Hz and 140 Hz. We further used the “find_bad_channels_maxwell” function from the MNE-Python software, plus visual inspection to identify bad channels, and further used the “maxwell_filter” function to implement bad channel reconstruction, movement compensation, and temporal spatiotemporal signal space separation. On average, five channels were interpolated per participant. To suppress eye movement and cardio artefacts, we used the SSP method to remove one eye and one cardio component as provided by the MNE-Python software.

The mean evoked responses (baseline-corrected) in the time domain were computed for each of the categories places (scenes) and faces, separately, with a window from −200 ms to 2500 ms after stimulus onset. The mean signals were then baseline-corrected with a window from −200 ms to 0 ms relative to stimulus onset.

The mean evoked responses (baseline corrected) in the time domain were used for source-space analysis at the surface level. Source estimation was performed using L2-minimum-norm estimation (depth weighting coefficients: 0.8, loose orientation constraint: 0.2) on an ico-5 source space (10242 sources per hemisphere). We used individual MRI images for head modelling. The MRI data were pre-processed in Freesurfer V.7.1.4 (Fischel, 2012), and the head model (1-layer boundary element model, BEM, with the default conductivity of 0.3) was created in MNE-Python. Source estimation was used with the standard ‘whitening’ approach implemented in the MNE-Python software to combine data from different sensor types.

The MEG data were converted into the HCP-MMP surface space^55^ using the MNE-Python functions ‘fetch_hcp_mmp_parcellation’ and ‘extract_label_time_course’ to produce for each participant a 1 ms MEG time-series in HCP-MMP space. As described previously^54^, although the spatial resolution of the MEG data may not be sufficient to provide an independent signal for each of the 360 cortical regions in the HCP-MMP atlas, the use of the atlas is potentially valuable because the cortical regions in the atlas are themselves well-defined^55^, and use of this atlas provides a framework for comparing findings from different investigations of the cortex^25,38,48,49,57,63–65,67–69,87,115–119^. Also, the spatial resolution of MEG may be poorer for visual cortical regions far from the skull, due to the inverse problem, though this is unlikely to account for the findings described here, for many regions high in the hierarchy such as the TE regions are as close to the skull as early visual cortical regions (see Figs. S1–5).

The effective connectivity between the 360 cortical regions was computed with the same Hopf generative effective connectivity algorithm used for the fMRI data^48^. This is important to note, for the use of the same algorithm that the directionality with the MEG and fMRI data can be compared. Because the MEG time series could be very long, the effective connectivity was calculated with an analytic version of the Hopf algorithm, rather than the simulation approach used for fMRI data^48^. The analytic approach is described in the Supplementary Material, and when tested with fMRI data, produced very similar results to the simulation approach (*r* > 0.95 for a comparison of the effective connectivities calculated with the simulation and analytic methods).

### Brain atlas and region selection

To construct the effective connectivity for the cortical regions of interest in this investigation with other cortical regions in the human brain, the parcellation of human cortical regions was used that is provided by the HCP-MMP which has 360 cortical regions^55^. The cortical regions in this parcellation^55^ are shown in Figs. 1 and S1, and a list of the cortical regions and the divisions into which they are placed are provided in Table S1 in the reordered form used in the extended volumetric HCPex atlas^120^.

The 30 visual cortical regions selected for connectivity analysis here were as follows, with reference to Fig. 1 useful in showing where these regions are in the human brain. The regions are grouped based on earlier evidence^48,55^ purely to simplify the description of the connectivity, and the groups are separated by red lines in Figs. 2–6.

Group 1: early visual cortical areas V1, V2, V3, and V4 of the HCP-MMP atlas.

Group 2: the retrosplenial group includes the ProStriate region and the DVT region which are retrosplenial regions in the HCP-MMP atlas where viewing scenes produces activations with fMRI which sometimes include POS1^25,33^.

Group 3: the ventromedial group VMV1–3 and VVC, and Group 4: the parahippocampal PHA1–3, are regions in the HCP-MMP atlas where viewing scenes produces activations with fMRI^25,33^ in what corresponds to the PPA^29,30^, which might better be termed the PSA because it is where the individual looks in scenes, not the place where the individual is located, that produces thise activations^6^.

Group 5: includes the Hipp and related regions including the EC and PeEc.

Group 6: includes the FFC, PIT and V8.

Group 7: TE1p and TE2p are the last mainly unimodal visual cortical regions, and correspond to the macaque inferior temporal visual cortex^48,54^.

Group 8: the anterior temporal lobe group includes TE1a, TE1m and TE2a, and the temporal pole regions TGd and TGv, which are multimodal semantic regions^48,54,63^.

### Measurement of effective connectivity

The effective connectivity between all pairs of the 360 cortical regions was computed with the same Hopf generative effective connectivity algorithm used for the fMRI data^48^, and the text that follows are similar to that used in some earlier descriptions, which helps to show that the same effective connectivity algorithm was used as in the earlier investigations^48,54,63,65,66,68,69,87^. Effective connectivity measures the effect of one brain region on another, and utilizes differences detected at different times in the signals in each connected pair of brain regions to infer the effects of one brain region on another. One such approach is dynamic causal modelling, but it applies most easily to activation studies, and is typically limited to measuring the effective connectivity between just a few brain areas^121–123^, though there have been moves to extend it to resting state studies and more brain areas^124,125^. The method used here in refs. ^57,64^ was developed from a Hopf algorithm to enable the measurement of effective connectivity between many brain areas, as described by Deco et al.^73^. A principle is that the functional connectivity is measured at time *t* and time *t* *+* *tau*, where *tau* was set to 20 ms.

To infer effective connectivity, we use a whole-brain model that allows us to analyse the MEG signal across all brain regions and time. We use the so-called Hopf computational model, which integrates the dynamics of Stuart–Landau oscillators, expressing the activity of each brain region, by the underlying anatomical connectivity^72^. As mentioned above, we include in the model 360 cortical brain areas^120^. The local dynamics of each brain area (node) is given by Stuart–Landau oscillators which express the normal form of a supercritical Hopf bifurcation, describing the transition from noisy to oscillatory dynamics^126^. During the last years, numerous studies have been able to show how the Hopf whole-brain model successfully simulates empirical electrophysiology^127,128^, MEG^129^, and fMRI^72,130–132^.

The Hopf whole-brain model can be expressed mathematically as follows:

1 $$\frac{d{x}_{i}}{{dt}}=\,\overbrace{\left[{a}_{i}-{x}_{i}^{2}-{y}_{i}^{2}\right]{x}_{i}-{\omega }_{i}{y}_{i}}^{{{Local}}\; {{dynamics}}}\,+\,\overbrace{G{\sum }_{j=1}^{N}{C}_{{ij}}\left({x}_{j}-{x}_{i}\right)}^{{Coupling}}\,+\overbrace{\beta {\eta }_{i}\left(t\right)}^{{Gaussiannoise}}$$

2 $$\frac{d{y}_{i}}{{dt}}=\,\left[{a}_{i}-{x}_{i}^{2}-{y}_{i}^{2}\right]{y}_{i}+{{{{\rm{\omega }}}}}_{i}{x}_{i}\,+{{{\rm{G}}}}{\sum }_{j=1}^{N}{C}_{{ij}}\left({y}_{j}-{y}_{i}\right)\,+{{{\rm{\beta }}}}{{{{\rm{\eta }}}}}_{i}\left(t\right)$$

Equations 1 and 2 describe the coupling of Stuart–Landau oscillators through an effective connectivity matrix C. The ${x}_{i}\left(t\right)$ term represents the simulated BOLD signal data of brain area *i*. The values of ${y}_{i}\left(t\right)$ are relevant to the dynamics of the system but are not part of the information read out from the system. These equations, ${{{{\rm{\eta }}}}}_{i}\left(t\right)$ provides additive Gaussian noise with standard deviation β. The Stuart–Landau oscillators for each brain area *i* express a Hopf normal form that has a supercritical bifurcation at ${a}_{i}$ = 0, so that if ${a}_{i}$ > 0 the system has a stable limit cycle with frequency ${f}_{i}$ = ${{{{\rm{\omega }}}}}_{i}$*/2π* (where ${{{{\rm{\omega }}}}}_{i}$ is the angular velocity); and when ${a}_{i}$ < 0 the system has a stable fixed point representing a low activity noisy state. The intrinsic frequencies are fitted from the data, as given by the averaged peak frequency of the narrowband BOLD signals of each brain region. The intrinsic frequency ${f}_{i}$ of each Stuart–Landau oscillator corresponding to a brain area *i* was in the 0.5–2 Hz band (*i* = 1, …, 360) for the HCP MEG data used here, which was sampled at 20 ms and not further filtered. The mean power spectrum across participants from the time series of the MEG signal for each of the 360 cortical regions used in the analyses described here is shown in Fig. S3. The coupling term representing the input received in node *i* from every other node *j*, is weighted by the corresponding effective connectivity ${C}_{{ij}}$. The coupling is the canonical diffusive coupling, which approximates the simplest (linear) part of a general coupling function. *G* denotes the global coupling weight, scaling equally the total input received in each brain area. While the oscillators are weakly coupled, the periodic orbit of the uncoupled oscillators is preserved.

The effective connectivity ($C$) matrix is derived by optimizing the conductivity of each connection in the matrix in order to fit the empirical functional connectivity (${{{{\boldsymbol{FC}}}}}^{{{\rm{empirical}}}}$) pairs and the lagged normalised covariance, the ${{{\boldsymbol{F}}}}{{{{\boldsymbol{S}}}}}^{{{\rm{empirical}}}}$ pairs. By this, we are able to infer a non-symmetric Effective Connectivity matrix (see ref. ^133^). We refer to this as a generative effective connectivity model approach because the $C$ matrix is used to generate the functional connectivity and lagged normalised covariance matrices, and the $C$ matrix is optimised so that the simulated matrices match the empirically measured matrices. Note that ${{{\boldsymbol{F}}}}{{{{\boldsymbol{S}}}}}^{{{\rm{empirical}}}}$, i.e. the normalised lagged covariance of the functional connectivity between pairs, lagged at $\tau$, breaks the symmetry and thus is fundamental for our purpose. Specifically, we compute the distance between the model functional connectivity ${{{{\boldsymbol{FC}}}}}^{{{\rm{model}}}}$ calculated analytically from the current estimate of the effective connectivity and the empirical data ${{{{\boldsymbol{FC}}}}}^{{{\rm{empirical}}}}$, as well as the calculated model ${{{{\boldsymbol{FS}}}}}^{{{\rm{model}}}}$ and empirical data ${{{\boldsymbol{F}}}}{{{{\boldsymbol{S}}}}}^{{{\rm{empirical}}}}$ and adjust each effective connection (entry in the effective connectivity matrix) separately with a gradient-descent approach. The model is run repeatedly with the updated effective connectivity until the fit converges towards a stable value.

We start with the anatomical connectivity obtained with probabilistic tractography from dMRI (or from an initial zero *C*_*ij*_ matrix) and use a pseudo gradient procedure for updating the effective connectivity matrix (see Eq. 11 in the Supplementary Material). The effective connectivity matrices shown here were those computed without the structural connection matrix, as use of the structural connectivity matrix limited the connectivity to fewer links than were otherwise found with these MEG data, probably because the DTI analysis missed some connections. However, the correlation between the matrices produced with these different methods was reasonable (0.80).

### Effective connectome

Whole-brain effective connectivity (*EC*) analysis was performed between the 30 visual cortical regions described above (see Fig. 1 and S1) and the 360 regions defined in the surface-based HCP-MMP atlas^55^ in their reordered form provided in Table S1, described in the Supplementary Material^120^. This *EC* was computed across all 21 participants, and the reliability was checked by a data split, which showed a correlation of 0.85 between the two halves. For each participant, the mean for the 2700-point long time series was calculated for each trial type (places/scenes and faces). From this, the functional connectivity *FC* for the 360 cortical regions and the covariance *COV* of the connectivity for the 360 cortical regions were calculated from the time series and the time series delayed by tau (where tau =20 ms) was calculated for each participant, and then the FC and COV matrices were averaged across participants. These provided the inputs *FC*^emp^ and *COV*^tauemp^ to the effective connectivity algorithm (*COV*^tauemp^ refers to the ${{{\boldsymbol{F}}}}{{{{\boldsymbol{S}}}}}^{{{\rm{empirical}}}}$ defined above.) Because effective connectivity measured in the way described utilises functional connectivity and what can be viewed as a lagged functional connectivity with a lag of tau, effective connectivity is not limited to measuring direct neuronal connections, but may reflect connectivity over perhaps 1–3 synapses (based on the evidence for example that V1 does not have effective connectivity with this approach with all visual cortical regions). We emphasize though that the generative effective connectivity algorithm is non-linear with respect to the functional connectivity, in that the generative effective connectivity algorithm sets to zero those connectivities that are not useful in generating the FC and COV matrices.

### Statistics and reproducibility

The key statistical analyses for the new investigation described here are on the directionality of the effective connectivities between key sets of cortical regions when scenes are being viewed, with the data illustrated in Fig. 3. Paired within-subjects comparisons were performed to test the differences in the effective connectivities in the two directions calculated for each participant. The degrees of freedom reflect the number of participants (21). The tests were two-tailed, and exact values were provided for the t and p values to provide evidence about the reliability of each test performed. Only a few preplanned tests were performed. Similar tests were performed for key cortical regions in the ventrolateral ‘What’ stream for faces and objects^34^ based on the data shown in Fig. 4 when faces were the stimuli. Further, the effective connectivity was computed across all 21 participants, and the reliability was checked by a data split, which showed a correlation of 0.85 between the two halves.

We note that the use of MEG is important, for the directionality of the effective connectivity when measured with resting-state fMRI for faces, places, tools and body parts shows as the reverse of what is expected and of what is found with MEG^48^, and this is probably related to the slow timecourse of fMRI which means that much of what is measured with fMRI resting state effective connectivity is the top–down effects from the top of the visual hierarchy where short-term memory keeps representations active^54^. All of our previous publications on effective connectivity measured with this algorithm with fMRI in different brain systems do show the effective connectivity in the direction found with MEG, and that would be expected for the initial flow of signal up through a sensory cortical hierarchy starting for example with V1 and progressing to the temporal lobe^48,49,57,63–65,67–69,87^.

### Functional connectivity

For comparison with the effective connectivity, the functional connectivity was also measured from the MEG signals with the identical set of participants and data. The functional connectivity was measured by the Pearson correlation between the MEG signal time series for each pair of brain regions, and is the *FC*^emp^ referred to above. A threshold was used for the presentation of the findings in Fig. S3, to set the sparseness of what is shown to a level commensurate with the effective connectivity, to facilitate comparison between the functional and the effective connectivity. The functional connectivity can provide evidence that may relate to interactions between brain regions, while providing no evidence about causal direction-specific effects. High functional connectivity may in this scenario thus reflect strong physiological interactions between areas, and provide a different type of evidence to effective connectivity. The effective connectivity is non-linearly related to the functional connectivity, with effective connectivities being identified (i.e. greater than zero) only for the links with relatively high functional connectivity.

### Connections shown with diffusion tractography

Diffusion tractography can provide evidence about fibre pathways linking different brain regions with a method that is completely different to the ways in which effective and functional connectivity are measured. Diffusion tractography shows only direct connections, so comparison with effective connectivity can help to suggest which effective connectivities may be mediated directly or indirectly. Diffusion tractography does not provide evidence about the direction of connections. Diffusion tractography was performed in a set of 171 HCP participants imaged at 7 T with methods described in detail elsewhere^56^. Some of the results are provided elsewhere^48,56^, but are shown in Fig. 5 for exactly the visual cortical regions investigated here with MEG, to facilitate comparison.

The major parameters were: 1.05 mm isotropic voxels; a two-shell acquisition scheme with b-values = 1000 s/mm^2^, 2000 s/mm^2^, repetition time/echo time = 7000/71 ms, 65 unique diffusion gradient directions and 6 b0 images obtained for each phase encoding direction pair (AP and PA pairs). Pre-processing steps included distortion correction, eddy-current correction, motion correction, and gradient non-linearity correction. In brief, whole-brain tractography was reconstructed for each subject in native space. To improve the tractography termination accuracy in GM, MRtrix3’s 5ttgen command was used to generate multi-tissue segment images (5tt) using T1 images, the segmented tissues were then co-registered with the b0 image in diffusion space. For multi-shell data, tissue response functions in GM, WM, and CSF were estimated by the MRtrix3’ dwi2response function with the Dhollander algorithm^134^. A multi-shell multi-tissue constrained spherical deconvolution model with lmax = 8 and prior co-registered 5tt image was used on the preprocessed multi-shell DWI data to obtain the fibre orientation distribution (FOD) function^135,136^. Based on the voxel-wise FOD, anatomically-constrained tractography using the probabilistic tracking algorithm: iFOD2 (2nd order integration based on FOD) with dynamic seeding was applied to generate the initial tractogram (1 million streamlines with maximum tract length = 250 mm and minimal tract length = 5 mm). To quantify the number of streamlines connecting pairs of regions, the updated version of the spherical-deconvolution informed filtering of the tractograms method was applied, which provides more biologically meaningful estimates of structural connection density^137^.

### Reporting summary

Further information on research design is available in the Nature Portfolio Reporting Summary linked to this article.

### Supplementary information

Supplemental Material

Reporting Summary

## Data Availability

The MEG data which are very large are available from the corresponding author on reasonable request.

## Code availability

Code for the Hopf generative effective connectivity algorithm is available at https://github.com/decolab/gec. Matlab 2023; Freesurfer V.7.1.4; and MNE-Python Version 1.5.1 were used.

## References

[1] Moscovitch, M., Cabeza, R., Winocur, G. & Nadel, L. Episodic memory and beyond: the hippocampus and neocortex in transformation. *Annu. Rev. Psychol.* **67**, 105–134 (2016). 26726963 10.1146/annurev-psych-113011-143733 PMC5060006

[2] Squire, L. R. & Wixted, J. T. The cognitive neuroscience of human memory since H.M. *Annu. Rev. Neurosci.* **34**, 259–288 (2011). 21456960 10.1146/annurev-neuro-061010-113720 PMC3192650

[3] Burgess, N., Jackson, A., Hartley, T. & O’Keefe, J. Predictions derived from modelling the hippocampal role in navigation. *Biol. Cybern.* **83**, 301–312 (2000). 11007303 10.1007/s004220000172

[4] O’Keefe, J., Burgess, N., Donnett, J. G., Jeffery, K. J. & Maguire, E. A. Place cells, navigational accuracy, and the human hippocampus. *Philos. Trans. R. Soc. B* **353**, 1333–1340 (1998). 10.1098/rstb.1998.0287 PMC1692339 9770226

[5] Burgess, N. & O’Keefe, J. Neuronal computations underlying the firing of place cells and their role in navigation. *Hippocampus* **6**, 749–762 (1996). 9034860 10.1002/(SICI)1098-1063(1996)6:6<749::AID-HIPO16>3.0.CO;2-0

[6] Rolls, E. T. Hippocampal spatial view cells for memory and navigation, and their underlying connectivity in humans. *Hippocampus* **33**, 533–572 (2023). 36070199 10.1002/hipo.23467 PMC10946493

[7] O’Keefe, J. A review of the hippocampal place cells. *Prog. Neurobiol.* **13**, 419–439 (1979). 396576 10.1016/0301-0082(79)90005-4

[8] Moser, E. I., Moser, M. B. & McNaughton, B. L. Spatial representation in the hippocampal formation: a history. *Nat. Neurosci.* **20**, 1448–1464 (2017). 29073644 10.1038/nn.4653

[9] Georges-François, P., Rolls, E. T. & Robertson, R. G. Spatial view cells in the primate hippocampus: allocentric view not head direction or eye position or place. *Cereb. Cortex* **9**, 197–212 (1999). 10355900 10.1093/cercor/9.3.197

[10] Rolls, E. T., Treves, A., Robertson, R. G., Georges-François, P. & Panzeri, S. Information about spatial view in an ensemble of primate hippocampal cells. *J. Neurophysiol.* **79**, 1797–1813 (1998). 9535949 10.1152/jn.1998.79.4.1797

[11] Robertson, R. G., Rolls, E. T. & Georges-François, P. Spatial view cells in the primate hippocampus: Effects of removal of view details. *J. Neurophysiol.* **79**, 1145–1156 (1998). 9497397 10.1152/jn.1998.79.3.1145

[12] Rolls, E. T., Robertson, R. G. & Georges-François, P. Spatial view cells in the primate hippocampus. *Eur. J. Neurosci.* **9**, 1789–1794 (1997). 9283835 10.1111/j.1460-9568.1997.tb01538.x

[13] Rolls, E. T. Hippocampal spatial view cells, place cells, and concept cells: view representations. *Hippocampus* **33**, 667–687 (2023). 37035903 10.1002/hipo.23536

[14] Rolls, E. T. et al. Hippocampal neurons in the monkey with activity related to the place in which a stimulus is shown. *J. Neurosci.* **9**, 1835–1845 (1989). 2723752 10.1523/JNEUROSCI.09-06-01835.1989 PMC6569734

[15] Rolls, E. T. & O’Mara, S. M. View-responsive neurons in the primate hippocampal complex. *Hippocampus* **5**, 409–424 (1995). 8773254 10.1002/hipo.450050504

[16] Wirth, S., Baraduc, P., Plante, A., Pinede, S. & Duhamel, J. R. Gaze-informed, task-situated representation of space in primate hippocampus during virtual navigation. *PLoS Biol.* **15**, e2001045 (2017). 28241007 10.1371/journal.pbio.2001045 PMC5328243

[17] Zhu, S. L., Lakshminarasimhan, K. J. & Angelaki, D. E. Computational cross-species views of the hippocampal formation. *Hippocampus* **33**, 586–599 (2023). 37038890 10.1002/hipo.23535 PMC10947336

[18] Mao, D. et al. Spatial modulation of hippocampal activity in freely moving macaques. *Neuron* **109**, 3521–3534.e3526 (2021). 34644546 10.1016/j.neuron.2021.09.032 PMC8571030

[19] Tsitsiklis, M. et al. Single-neuron representations of spatial targets in humans. *Curr. Biol.* **30**, 245–253.e244 (2020). 31902728 10.1016/j.cub.2019.11.048 PMC6981010

[20] Donoghue, T. et al. Single neurons in the human medial temporal lobe flexibly shift representations across spatial and memory tasks. *Hippocampus* **33**, 600–615 (2023). 37060325 10.1002/hipo.23539 PMC10231142

[21] Qasim, S. E. et al. Memory retrieval modulates spatial tuning of single neurons in the human entorhinal cortex. *Nat. Neurosci.* **22**, 2078–2086 (2019). 31712776 10.1038/s41593-019-0523-z PMC6897360

[22] Qasim, S. E., Fried, I. & Jacobs, J. Phase precession in the human hippocampus and entorhinal cortex. *Cell* **184**, 3242–3255.e3210 (2021). 33979655 10.1016/j.cell.2021.04.017 PMC8195854

[23] Ison, M. J., Quian Quiroga, R. & Fried, I. Rapid encoding of new memories by individual neurons in the human brain. *Neuron* **87**, 220–230 (2015). 26139375 10.1016/j.neuron.2015.06.016 PMC4509714

[24] Piza, D. B. et al. Primacy of vision shapes behavioral strategies and neural substrates of spatial navigation in marmoset hippocampus. *Nat. Commun.* **15**, 4053 (2024). 38744848 10.1038/s41467-024-48374-2 PMC11093997

[25] Sulpizio, V., Galati, G., Fattori, P., Galletti, C. & Pitzalis, S. A common neural substrate for processing scenes and egomotion-compatible visual motion. *Brain Struct. Funct.* **225**, 2091–2110 (2020). 32647918 10.1007/s00429-020-02112-8 PMC7473967

[26] Natu, V. S. et al. Sulcal depth in the medial ventral temporal cortex predicts the location of a place-selective region in macaques, children, and adults. *Cereb. Cortex* **31**, 48–61 (2021). 32954410 10.1093/cercor/bhaa203 PMC7727388

[27] Kamps, F. S., Julian, J. B., Kubilius, J., Kanwisher, N. & Dilks, D. D. The occipital place area represents the local elements of scenes. *Neuroimage* **132**, 417–424 (2016). 26931815 10.1016/j.neuroimage.2016.02.062 PMC4872505

[28] Epstein, R. & Kanwisher, N. A cortical representation of the local visual environment. *Nature* **392**, 598–601 (1998). 9560155 10.1038/33402

[29] Epstein, R. A. & Baker, C. I. Scene perception in the human brain. *Annu Rev. Vis. Sci.* **5**, 373–397 (2019). 31226012 10.1146/annurev-vision-091718-014809 PMC6989029

[30] Epstein, R. A. & Julian, J. B. Scene areas in humans and macaques. *Neuron* **79**, 615–617 (2013). 23972591 10.1016/j.neuron.2013.08.001 PMC3800114

[31] Epstein, R. A. Parahippocampal and retrosplenial contributions to human spatial navigation. *Trends Cogn. Sci.* **12**, 388–396 (2008). 18760955 10.1016/j.tics.2008.07.004 PMC2858632

[32] Epstein, R. The cortical basis of visual scene processing. *Vis. Cogn.* **12**, 954–978 (2005). 10.1080/13506280444000607

[33] Rolls, E. T., Feng, J. & Zhang, R. Selective activations and functional connectivities to the sight of faces, scenes, body parts and tools in visual and non-visual cortical regions leading to the human hippocampus. *Brain Struct. Funct.* **229**, 1471–1493 (2024). 38839620 10.1007/s00429-024-02811-6 PMC11176242

[34] Rolls, E. T. Two what, two where, visual cortical streams in humans. *Neurosci. Biobehav. Rev.* **160**, 105650 (2024). 38574782 10.1016/j.neubiorev.2024.105650

[35] Rolls, E. T. Neurons including hippocampal spatial view cells, and navigation in primates including humans. *Hippocampus* **31**, 593–611 (2021). 33760309 10.1002/hipo.23324

[36] Rolls, E. T. Hippocampal discoveries: spatial view cells, and computations for memory and navigation, in primates including humans. *Hippocampus* (2024). 10.1002/hipo.23324 33760309

[37] Stringer, S. M., Rolls, E. T. & Trappenberg, T. P. Self-organizing continuous attractor network models of hippocampal spatial view cells. *Neurobiol. Learn. Mem.* **83**, 79–92 (2005). 15607692 10.1016/j.nlm.2004.08.003

[38] Rolls, E. T. *Brain Computations and Connectivity* (Oxford University Press, 2023).

[39] Rolls, E. T. & Wirth, S. Spatial representations in the primate hippocampus, and their functions in memory and navigation. *Prog. Neurobiol.* **171**, 90–113 (2018). 30219248 10.1016/j.pneurobio.2018.09.004

[40] Rolls, E. T. The memory systems of the human brain and generative artificial intelligence. *Heliyon* **10**, e31965 (2024). 38841455 10.1016/j.heliyon.2024.e31965 PMC11152951

[41] Maguire, E. A. The retrosplenial contribution to human navigation: a review of lesion and neuroimaging findings. *Scand. J. Psychol.* **42**, 225–238 (2001). 11501737 10.1111/1467-9450.00233

[42] Silson, E. H., Steel, A. D. & Baker, C. I. Scene-selectivity and retinotopy in medial parietal cortex. *Front. Hum. Neurosci.* **10**, 412 (2016). 27588001 10.3389/fnhum.2016.00412 PMC4988988

[43] Dilks, D. D., Julian, J. B., Paunov, A. M. & Kanwisher, N. The occipital place area is causally and selectively involved in scene perception. *J. Neurosci.* **33**, 1331–1336a (2013). 23345209 10.1523/JNEUROSCI.4081-12.2013 PMC3711611

[44] Hasson, U., Harel, M., Levy, I. & Malach, R. Large-scale mirror-symmetry organization of human occipito-temporal object areas. *Neuron* **37**, 1027–1041 (2003). 12670430 10.1016/S0896-6273(03)00144-2

[45] Baldassano, C., Esteva, A., Fei-Fei, L. & Beck, D. M. Two distinct scene-processing networks connecting vision and memory. *eNeuro* **3**, e0178–0116.2016 (2016). 10.1523/ENEURO.0178-16.2016 PMC5075944 27822493

[46] Nasr, S., Devaney, K. J. & Tootell, R. B. Spatial encoding and underlying circuitry in scene-selective cortex. *Neuroimage* **83**, 892–900 (2013). 23872156 10.1016/j.neuroimage.2013.07.030 PMC3815999

[47] Watson, D. M. & Andrews, T. J. Mapping the functional and structural connectivity of the scene network. *Hum. Brain Mapp.* **45**, e26628 (2024). 38376190 10.1002/hbm.26628 PMC10878195

[48] Rolls, E. T., Deco, G., Huang, C.-C. & Feng, J. Multiple cortical visual streams in humans. *Cereb. Cortex* **33**, 3319–3349 (2023). 35834308 10.1093/cercor/bhac276

[49] Rolls, E. T., Wirth, S., Deco, G., Huang, C.-C. & Feng, J. The human posterior cingulate, retrosplenial and medial parietal cortex effective connectome, and implications for memory and navigation. *Hum. Brain Mapp.* **44**, 629–655 (2023). 36178249 10.1002/hbm.26089 PMC9842927

[50] Libby, L. A., Ekstrom, A. D., Ragland, J. D. & Ranganath, C. Differential connectivity of perirhinal and parahippocampal cortices within human hippocampal subregions revealed by high-resolution functional imaging. *J. Neurosci.* **32**, 6550–6560 (2012). 22573677 10.1523/JNEUROSCI.3711-11.2012 PMC3374643

[51] Steel, A., Billings, M. M., Silson, E. H. & Robertson, C. E. A network linking scene perception and spatial memory systems in posterior cerebral cortex. *Nat. Commun.* **12**, 2632 (2021). 33976141 10.1038/s41467-021-22848-z PMC8113503

[52] Kahn, I., Andrews-Hanna, J. R., Vincent, J. L., Snyder, A. Z. & Buckner, R. L. Distinct cortical anatomy linked to subregions of the medial temporal lobe revealed by intrinsic functional connectivity. *J. Neurophysiol.* **100**, 129–139 (2008). 18385483 10.1152/jn.00077.2008 PMC2493488

[53] Reznik, D., Trampel, R., Weiskopf, N., Witter, M. P. & Doeller, C. F. Dissociating distinct cortical networks associated with subregions of the human medial temporal lobe using precision neuroimaging. *Neuron* **111**, 2756–2772.e2757 (2023). 37390820 10.1016/j.neuron.2023.05.029

[54] Rolls, E. T., Deco, G., Zhang, Y. & Feng, J. Hierarchical organization of the human ventral visual streams revealed with magnetoencephalography. *Cereb. Cortex* **33**, 10686–10701 (2023). 37689834 10.1093/cercor/bhad318

[55] Glasser, M. F. et al. A multi-modal parcellation of human cerebral cortex. *Nature* **536**, 171–178 (2016). 27437579 10.1038/nature18933 PMC4990127

[56] Huang, C.-C., Rolls, E. T., Hsu, C.-C. H., Feng, J. & Lin, C.-P. Extensive cortical connectivity of the human hippocampal memory system: beyond the “what” and “where” dual-stream model. *Cereb. Cortex* **31**, 4652–4669 (2021). 34013342 10.1093/cercor/bhab113 PMC8866812

[57] Rolls, E. T., Deco, G., Huang, C. C. & Feng, J. The effective connectivity of the human hippocampal memory system. *Cereb. Cortex* **32**, 3706–3725 (2022). 35034120 10.1093/cercor/bhab442

[58] Larson-Prior, L. J. et al. Adding dynamics to the human connectome project with MEG. *Neuroimage* **80**, 190–201 (2013). 23702419 10.1016/j.neuroimage.2013.05.056 PMC3784249

[59] Barch, D. M. et al. Function in the human connectome: task-fMRI and individual differences in behavior. *Neuroimage* **80**, 169–189 (2013). 23684877 10.1016/j.neuroimage.2013.05.033 PMC4011498

[60] Yokoyama, C. et al. Comparative connectomics of the primate social brain. *Neuroimage* **245**, 118693 (2021). 34732327 10.1016/j.neuroimage.2021.118693 PMC9159291

[61] Van Essen, D. C. & Glasser, M. F. Parcellating cerebral cortex: how invasive animal studies inform noninvasive mapmaking in humans. *Neuron* **99**, 640–663 (2018). 30138588 10.1016/j.neuron.2018.07.002 PMC6149530

[62] Colclough, G. L. et al. The heritability of multi-modal connectivity in human brain activity. *Elife* **6**, e20178 (2017). 28745584 10.7554/eLife.20178 PMC5621837

[63] Rolls, E. T., Deco, G., Huang, C.-C. & Feng, J. The human language effective connectome. *Neuroimage* **258**, 119352 (2022). 35659999 10.1016/j.neuroimage.2022.119352

[64] Rolls, E. T., Deco, G., Huang, C. C. & Feng, J. The human orbitofrontal cortex, vmPFC, and anterior cingulate cortex effective connectome: emotion, memory, and action. *Cereb. Cortex* **33**, 330–356 (2022). 35233615 10.1093/cercor/bhac070

[65] Rolls, E. T., Deco, G., Huang, C. C. & Feng, J. The human posterior parietal cortex: effective connectome, and its relation to function. *Cereb. Cortex* **33**, 3142–3170 (2023). 35834902 10.1093/cercor/bhac266 PMC10401905

[66] Ma, Q., Rolls, E. T., Huang, C.-C., Cheng, W. & Feng, J. Extensive cortical functional connectivity of the human hippocampal memory system. *Cortex* **147**, 83–101 (2022). 35026557 10.1016/j.cortex.2021.11.014

[67] Rolls, E. T., Deco, G., Huang, C.-C. & Feng, J. Human amygdala compared to orbitofrontal cortex connectivity, and emotion. *Prog. Neurobiol.* **220**, 102385 (2023). 36442728 10.1016/j.pneurobio.2022.102385

[68] Rolls, E. T., Deco, G., Huang, C. C. & Feng, J. Prefrontal and somatosensory-motor cortex effective connectivity in humans. *Cereb. Cortex* **33**, 4939–4963 (2023). 36227217 10.1093/cercor/bhac391

[69] Rolls, E. T., Rauschecker, J. P., Deco, G., Huang, C. C. & Feng, J. Auditory cortical connectivity in humans. *Cereb. Cortex* **33**, 6207–6227 (2023). 36573464 10.1093/cercor/bhac496 PMC10422925

[70] Baker, C. M. et al. A connectomic atlas of the human cerebrum-chapter 7: the lateral parietal lobe. *Oper. Neurosurg.* **15**, S295–S349 (2018). 10.1093/ons/opy261 PMC6887702 30260428

[71] Baker, C. M. et al. A connectomic atlas of the human cerebrum-chapter 6: the temporal lobe. *Oper. Neurosurg.* **15**, S245–S294 (2018). 10.1093/ons/opy260 PMC6887748 30260447

[72] Deco, G., Kringelbach, M. L., Jirsa, V. K. & Ritter, P. The dynamics of resting fluctuations in the brain: metastability and its dynamical cortical core. *Sci. Rep.* **7**, 3095 (2017). 28596608 10.1038/s41598-017-03073-5 PMC5465179

[73] Deco, G. et al. Awakening: predicting external stimulation to force transitions between different brain states. *Proc. Natl. Acad. Sci.* **116**, 18088–18097 (2019). 31427539 10.1073/pnas.1905534116 PMC6731634

[74] Wallis, G. & Rolls, E. T. Invariant face and object recognition in the visual system. *Prog. Neurobiol.* **51**, 167–194 (1997). 9247963 10.1016/S0301-0082(96)00054-8

[75] Panzeri, S., Rolls, E. T., Battaglia, F. & Lavis, R. Speed of feedforward and recurrent processing in multilayer networks of integrate-and-fire neurons. *Network* **12**, 423–440 (2001). 11762898 10.1080/net.12.4.423.440

[76] Rolls, E. T. *Cerebral Cortex: Principles of Operation* (Oxford University Press, 2016).

[77] Battaglia, F. P. & Treves, A. Stable and rapid recurrent processing in realistic auto-associative memories. *Neural Comput.* **10**, 431–450 (1998). 9472489 10.1162/089976698300017827

[78] Rolls, E. T. & Turova, T. S. Visual cortical networks for ‘What’ and ‘Where’ to the human hippocampus revealed with dynamical graphs (2024). 10.1093/cercor/bhaf106 40347158

[79] Rolls, E. T. Neurophysiological mechanisms underlying face processing within and beyond the temporal cortical visual areas. *Philos. Trans. R. Soc. Lond. B* **335**, 11–21 (1992). 1348130 10.1098/rstb.1992.0002

[80] Baylis, G. C., Rolls, E. T. & Leonard, C. M. Functional subdivisions of the temporal lobe neocortex. *J. Neurosci.* **7**, 330–342 (1987). 3819816 10.1523/JNEUROSCI.07-02-00330.1987 PMC6568924

[81] Hasselmo, M. E., Rolls, E. T., Baylis, G. C. & Nalwa, V. Object-centred encoding by face-selective neurons in the cortex in the superior temporal sulcus of the monkey. *Exp. Brain Res.* **75**, 417–429 (1989). 2721619 10.1007/BF00247948

[82] Hasselmo, M. E., Rolls, E. T. & Baylis, G. C. The role of expression and identity in the face-selective responses of neurons in the temporal visual cortex of the monkey. *Behav. Brain Res.* **32**, 203–218 (1989). 2713076 10.1016/S0166-4328(89)80054-3

[83] Pitcher, D. & Ungerleider, L. G. Evidence for a third visual pathway specialized for social perception. *Trends Cogn. Sci.* **25**, 100–110 (2021). 33334693 10.1016/j.tics.2020.11.006 PMC7811363

[84] Scherf, K. S., Behrmann, M., Humphreys, K. & Luna, B. Visual category-selectivity for faces, places and objects emerges along different developmental trajectories. *Dev. Sci.* **10**, F15–F30 (2007). 17552930 10.1111/j.1467-7687.2007.00595.x

[85] Kanwisher, N., McDermott, J. & Chun, M. M. The fusiform face area: a module in human extrastriate cortex specialized for face perception. *J. Neurosci.* **17**, 4302–4311 (1997). 9151747 10.1523/JNEUROSCI.17-11-04302.1997 PMC6573547

[86] Rolls, E. T. The hippocampus, ventromedial prefrontal cortex, and episodic and semantic memory. *Prog. Neurobiol.* **217**, 102334 (2022). 35870682 10.1016/j.pneurobio.2022.102334

[87] Rolls, E. T., Deco, G., Huang, C. C. & Feng, J. The connectivity of the human frontal pole cortex, and a theory of its involvement in exploit versus explore. *Cereb. Cortex* **34**, 1–19 (2024). 37991264 10.1093/cercor/bhad416

[88] Rolls, E. T. & Xiang, J.-Z. Spatial view cells in the primate hippocampus, and memory recall. *Rev. Neurosci.* **17**, 175–200 (2006). 16703951 10.1515/REVNEURO.2006.17.1-2.175

[89] Rolls, E. T., Xiang, J.-Z. & Franco, L. Object, space and object-space representations in the primate hippocampus. *J. Neurophysiol.* **94**, 833–844 (2005). 15788523 10.1152/jn.01063.2004

[90] Rolls, E. T. & Xiang, J.-Z. Reward-spatial view representations and learning in the hippocampus. *J. Neurosci.* **25**, 6167–6174 (2005). 15987946 10.1523/JNEUROSCI.1481-05.2005 PMC6725063

[91] Rolls, E. T., Zhang, R., Deco, G., Vatansever, D. & Feng, J. Selective brain activations and connectivities related to the storage and recall of human object-location, reward-location, and word-pair episodic memories. *Hum. Brain Mapp*. (2024). 10.1002/hbm.70056 PMC11494686 39436048

[92] McNaughton, B. L. et al. Deciphering the hippocampal polyglot: the hippocampus as a path integration system. *J. Exp. Biol.* **199**, 173–185 (1996). 8576689 10.1242/jeb.199.1.173

[93] Jiang, R., Andolina, I. M., Li, M. & Tang, S. Clustered functional domains for curves and corners in cortical area V4. *Elife* **10**, e63798 (2021). 33998459 10.7554/eLife.63798 PMC8175081

[94] Kim, T., Bair, W. & Pasupathy, A. Neural coding for shape and texture in macaque area V4. *J. Neurosci.* **39**, 4760–4774 (2019). 30948478 10.1523/JNEUROSCI.3073-18.2019 PMC6561689

[95] Rolls, E. T. Spatial coordinate transforms linking the allocentric hippocampal and egocentric parietal primate brain systems for memory, action in space, and navigation. *Hippocampus* **30**, 332–353 (2020). 31697002 10.1002/hipo.23171

[96] Rolls, E. T. & Treves, A. A theory of hippocampal function: new developments. *Prog. Neurobiol.* **238**, 102636 (2024). 38834132 10.1016/j.pneurobio.2024.102636

[97] Rolls, E. T., Zhang, C. & Feng, J. Hippocampal storage and recall of neocortical ‘What’–‘Where’ representations. *Hippocampus*10.1002/hipo.23636 (2024). 10.1002/hipo.23636 39221708

[98] Haak, K. V. & Beckmann, C. F. Objective analysis of the topological organization of the human cortical visual connectome suggests three visual pathways. *Cortex* **98**, 73–83 (2018). 28457575 10.1016/j.cortex.2017.03.020 PMC5780302

[99] De Araujo, I. E. T., Rolls, E. T. & Stringer, S. M. A view model which accounts for the spatial fields of hippocampal primate spatial view cells and rat place cells. *Hippocampus* **11**, 699–706 (2001). 11811664 10.1002/hipo.1085

[100] Rolls, E. T. A theory and model of scene representations with hippocampal spatial view cells (2024). 10.1002/hipo.70013 PMC12038316 40296500

[101] Rolls, E. T. Learning invariant object and spatial view representations in the brain using slow unsupervised learning. *Front. Comput. Neurosci.* **15**, 686239 (2021). 34366818 10.3389/fncom.2021.686239 PMC8335547

[102] Hubel, D. H. & Wiesel, T. N. Ferrier lecture. Functional architecture of macaque monkey visual cortex. *Proc. R. Soc. Lond. B. Biol. Sci.* **198**, 1–59 (1977). 20635 10.1098/rspb.1977.0085

[103] Wei, H., Dong, Z. & Wang, L. V4 shape features for contour representation and object detection. *Neural Netw.* **97**, 46–61 (2018). 29080474 10.1016/j.neunet.2017.09.010

[104] Nandy, A. S., Sharpee, T. O., Reynolds, J. H. & Mitchell, J. F. The fine structure of shape tuning in area V4. *Neuron* **78**, 1102–1115 (2013). 23791199 10.1016/j.neuron.2013.04.016 PMC3694358

[105] Roe, A. W. et al. Toward a unified theory of visual area V4. *Neuron* **74**, 12–29 (2012). 22500626 10.1016/j.neuron.2012.03.011 PMC4912377

[106] Feigenbaum, J. D. & Rolls, E. T. Allocentric and egocentric spatial information processing in the hippocampal formation of the behaving primate. *Psychobiology* **19**, 21–40 (1991). 10.1007/BF03337953

[107] Nasr, S. et al. Scene-selective cortical regions in human and nonhuman primates. *J. Neurosci.* **31**, 13771–13785 (2011). 21957240 10.1523/JNEUROSCI.2792-11.2011 PMC3489186

[108] Elshout, J. A., van den Berg, A. V. & Haak, K. V. Human V2A: a map of the peripheral visual hemifield with functional connections to scene-selective cortex. *J. Vis.* **18**, 22 (2018). 30267074 10.1167/18.9.22 PMC6159387

[109] Yu, H. H., Chaplin, T. A., Davies, A. J., Verma, R. & Rosa, M. G. A specialized area in limbic cortex for fast analysis of peripheral vision. *Curr. Biol.* **22**, 1351–1357 (2012). 22704993 10.1016/j.cub.2012.05.029

[110] Mikellidou, K. et al. Area prostriata in the human brain. *Curr. Biol.* **27**, 3056–3060.e3053 (2017). 28966090 10.1016/j.cub.2017.08.065

[111] Solomon, S. G. & Rosa, M. G. A simpler primate brain: the visual system of the marmoset monkey. *Front. Neural. Circuits* **8**, 96 (2014). 25152716 10.3389/fncir.2014.00096 PMC4126041

[112] Snyder, L. H., Grieve, K. L., Brotchie, P. & Andersen, R. A. Separate body- and world-referenced representations of visual space in parietal cortex. *Nature* **394**, 887–891 (1998). 9732870 10.1038/29777

[113] Rolls, E. T. Invariant visual object and face recognition: neural and computational bases, and a model, VisNet. *Front. Comput. Neurosci.* **6**, 1–70 (2012). 22723777 10.3389/fncom.2012.00035 PMC3378046

[114] Gramfort, A. et al. MEG and EEG data analysis with MNE-Python. *Front. Neurosci.* **7**, 267 (2013). 24431986 10.3389/fnins.2013.00267 PMC3872725

[115] Rolls, E. T., Feng, R., Cheng, W. & Feng, J. Orbitofrontal cortex connectivity is associated with food reward and body weight in humans. *Soc. Cogn. Affect. Neurosci.* **18**, nsab083 (2023). 34189586 10.1093/scan/nsab083 PMC10498940

[116] Wan, Z., Rolls, E. T., Cheng, W. & Feng, J. Brain functional connectivities that mediate the association between childhood traumatic events and adult mental health and cognition. *EBioMedicine* **79**, 104002 (2022). 35472671 10.1016/j.ebiom.2022.104002 PMC9058958

[117] Zhang, R., Rolls, E. T., Cheng, W. & Feng, J. Different cortical connectivities in human females and males relate to differences in strength and body composition, reward and emotional systems, and memory. *Brain Struct. Funct.* **229**, 47–61 (2024). 37861743 10.1007/s00429-023-02720-0 PMC10827883

[118] Rolls, E. T., Feng, R. & Feng, J. Lifestyle risks associated with brain functional connectivity and structure. *Hum. Brain Mapp.* **44**, 2479–2492 (2023). 36799566 10.1002/hbm.26225 PMC10028639

[119] Rolls, E. T. Emotion, motivation, decision-making, the orbitofrontal cortex, anterior cingulate cortex, and the amygdala. *Brain Struct. Funct.* **228**, 1201–1257 (2023). 37178232 10.1007/s00429-023-02644-9 PMC10250292

[120] Huang, C. C., Rolls, E. T., Feng, J. & Lin, C. P. An extended human connectome project multimodal parcellation atlas of the human cortex and subcortical areas. *Brain Struct. Funct.* **227**, 763–778 (2022). 34791508 10.1007/s00429-021-02421-6

[121] Valdes-Sosa, P. A., Roebroeck, A., Daunizeau, J. & Friston, K. Effective connectivity: influence, causality and biophysical modeling. *Neuroimage* **58**, 339–361 (2011). 21477655 10.1016/j.neuroimage.2011.03.058 PMC3167373

[122] Bajaj, S., Adhikari, B. M., Friston, K. J. & Dhamala, M. Bridging the gap: dynamic causal modeling and granger causality analysis of resting state functional magnetic resonance imaging. *Brain Connect.* **6**, 652–661 (2016). 27506256 10.1089/brain.2016.0422

[123] Friston, K. Causal modelling and brain connectivity in functional magnetic resonance imaging. *PLoS Biol.* **7**, e33 (2009). 19226186 10.1371/journal.pbio.1000033 PMC2642881

[124] Razi, A. et al. Large-scale DCMs for resting-state fMRI. *Netw. Neurosci.* **1**, 222–241 (2017). 29400357 10.1162/NETN_a_00015 PMC5796644

[125] Frassle, S. et al. Regression DCM for fMRI. *Neuroimage* **155**, 406–421 (2017). 28259780 10.1016/j.neuroimage.2017.02.090

[126] Kuznetsov, Y. A. (ed) *Elements of applied bifurcation theory* (Springer Science and Business Media, 2013).

[127] Freyer, F. et al. Biophysical mechanisms of multistability in resting-state cortical rhythms. *J. Neurosci.* **31**, 6353–6361 (2011). 21525275 10.1523/JNEUROSCI.6693-10.2011 PMC6622680

[128] Freyer, F., Roberts, J. A., Ritter, P. & Breakspear, M. A canonical model of multistability and scale-invariance in biological systems. *PLoS Comput. Biol.* **8**, e1002634 (2012). 22912567 10.1371/journal.pcbi.1002634 PMC3415415

[129] Deco, G. et al. Single or multiple frequency generators in on-going brain activity: a mechanistic whole-brain model of empirical MEG data. *Neuroimage* **152**, 538–550 (2017). 28315461 10.1016/j.neuroimage.2017.03.023 PMC5440176

[130] Kringelbach, M. L., McIntosh, A. R., Ritter, P., Jirsa, V. K. & Deco, G. The rediscovery of slowness: exploring the timing of cognition. *Trends Cogn. Sci.* **19**, 616–628 (2015). 26412099 10.1016/j.tics.2015.07.011

[131] Kringelbach, M. L. & Deco, G. Brain states and transitions: insights from computational neuroscience. *Cell Rep.* **32**, 108128 (2020). 32905760 10.1016/j.celrep.2020.108128

[132] Kringelbach, M. L., Perl, Y. S., Tagliazucchi, E. & Deco, G. Toward naturalistic neuroscience: Mechanisms underlying the flattening of brain hierarchy in movie-watching compared to rest and task. *Sci. Adv.* **9**, eade6049 (2023). 36638163 10.1126/sciadv.ade6049 PMC9839335

[133] Gilson, M., Moreno-Bote, R., Ponce-Alvarez, A., Ritter, P. & Deco, G. Estimation of directed effective connectivity from fMRI functional connectivity hints at asymmetries in the cortical connectome. *PLoS Comput. Biol.* **12**, e1004762 (2016). 26982185 10.1371/journal.pcbi.1004762 PMC4794215

[134] Dhollander, T., Raffelt, D. & Connelly, A. Unsupervised 3-tissue response function estimation from single-shell or multi-shell diffusion MR data without a co-registered T1 image. In *ISMRM Workshop on Breaking the Barriers of Diffusion MRI* 5 (ISMRM, Lisbon, 2016).

[135] Jeurissen, B., Tournier, J. D., Dhollander, T., Connelly, A. & Sijbers, J. Multi-tissue constrained spherical deconvolution for improved analysis of multi-shell diffusion MRI data. *Neuroimage* **103**, 411–426 (2014). 25109526 10.1016/j.neuroimage.2014.07.061

[136] Smith, S. M. Fast robust automated brain extraction. *Hum. Brain Mapp.* **17**, 143–155 (2002). 12391568 10.1002/hbm.10062 PMC6871816

[137] Smith, R. E., Tournier, J. D., Calamante, F. & Connelly, A. SIFT2: Enabling dense quantitative assessment of brain white matter connectivity using streamlines tractography. *Neuroimage* **119**, 338–351 (2015). 26163802 10.1016/j.neuroimage.2015.06.092

