# Supplementary material for: A ventromedial visual cortical ‘Where’ stream to the human hippocampus for spatial scenes revealed with magnetoencephalography
Source: Commun Biol. 2024 Aug 25;7:1047. doi: 10.1038/s42003-024-06719-z (PMC11345434; doi:10.1038/s42003-024-06719-z)
Supplement: Supplementary file 2 — Reporting Summary [file 42003_2024_6719_MOESM2_ESM.pdf]

nature portfolio

Corresponding author(s): E T Rolls

Last updated by author(s): Mar 29, 2024

Reporting Summary

Nature Portfolio wishes to improve the reproducibility of the work that we publish. This form provides structure for consistency and transparency in reporting. For further information on Nature Portfolio policies, see our Editorial Policies and the Editorial Policy Checklist.

Statistics

For all statistical analyses, confirm that the following items are present in the figure legend, table legend, main text, or Methods section.

n/a	Confirmed	
☐	☒	The exact sample size (*n*) for each experimental group/condition, given as a discrete number and unit of measurement
☒	☐	A statement on whether measurements were taken from distinct samples or whether the same sample was measured repeatedly
☐	☒	The statistical test(s) used AND whether they are one- or two-sided<br>*Only common tests should be described solely by name; describe more complex techniques in the Methods section.*
☒	☐	A description of all covariates tested
☒	☐	A description of any assumptions or corrections, such as tests of normality and adjustment for multiple comparisons
☒	☐	A full description of the statistical parameters including central tendency (e.g. means) or other basic estimates (e.g. regression coefficient) AND variation (e.g. standard deviation) or associated estimates of uncertainty (e.g. confidence intervals)
☐	☒	For null hypothesis testing, the test statistic (e.g. *F*, *t*, *r*) with confidence intervals, effect sizes, degrees of freedom and *P* value noted<br>*Give P values as exact values whenever suitable.*
☒	☐	For Bayesian analysis, information on the choice of priors and Markov chain Monte Carlo settings
☒	☐	For hierarchical and complex designs, identification of the appropriate level for tests and full reporting of outcomes
☒	☐	Estimates of effect sizes (e.g. Cohen's *d*, Pearson's *r*), indicating how they were calculated

Our web collection on statistics for biologists contains articles on many of the points above.

Software and code

Policy information about availability of computer code

Data collection	No code used for the data collection.
Data analysis	Matlab 2023; Freesurfer V.7.1.4; MNE-Python Version 1.5.1 ;

For manuscripts utilizing custom algorithms or software that are central to the research but not yet described in published literature, software must be made available to editors and reviewers. We strongly encourage code deposition in a community repository (e.g. GitHub). See the Nature Portfolio guidelines for submitting code & software for further information.

Data

Policy information about availability of data

All manuscripts must include a data availability statement. This statement should provide the following information, where applicable:

- Accession codes, unique identifiers, or web links for publicly available datasets
- A description of any restrictions on data availability
- For clinical datasets or third party data, please ensure that the statement adheres to our policy

Basic code for the Hopf generative effective connectivity algorithm is available at https://github.com/decolab/gec . The preprocessed MEG data are available on request.

Research involving human participants, their data, or biological material

Policy information about studies with human participants or human data. See also policy information about sex, gender (identity/presentation), and sexual orientation and race, ethnicity and racism.

Reporting on sex and gender	The number of female and male participants is stated.
Reporting on race, ethnicity, or other socially relevant groupings	The neuroimaging was performed with Chinese participants from Fudan University.
Population characteristics	Twenty-four participants from Fudan University aged 19-30 years (13 females) participated in the experiment.
Recruitment	By advertisement within Fudan University.
Ethics oversight	The study received ethical approval from the Ethics Committee of the Institute of Science and Technology for Brain Inspired Intelligence at Fudan University (reference number AF/SC-115/20230822).

Note that full information on the approval of the study protocol must also be provided in the manuscript.

Field-specific reporting

Please select the one below that is the best fit for your research. If you are not sure, read the appropriate sections before making your selection.

☒ Life sciences ☐ Behavioural & social sciences ☐ Ecological, evolutionary & environmental sciences

For a reference copy of the document with all sections, see nature.com/documents/nr-reporting-summary-flat.pdf

Life sciences study design

All studies must disclose on these points even when the disclosure is negative.

Sample size	21 participants is typical for MEG and fMRI investigations, and enabled statistically significant effects to be obtained.
Data exclusions	Twenty-four participants from Fudan University aged 19-30 years (13 females) participated in the experiment. Three of them had to be excluded because of MEG artefacts in the data, leaving 21 datasets for the final analysis
Replication	The results obtained are consistent with a previous paper (Rolls, E. T., Deco,G., Zhang,Y. and Feng,J. (2023) Hierarchical organisation of the human ventral visual streams revealed with magnetoencephalography. Cerebral Cortex 33: 10686-10701. doi: 10.1093/cercor/bhad318), but the results here are new in that scenes were used as the stimuli and the participants were different.
Randomization	The participants were not in different groups.
Blinding	The participants were not in different groups.

Reporting for specific materials, systems and methods

We require information from authors about some types of materials, experimental systems and methods used in many studies. Here, indicate whether each material, system or method listed is relevant to your study. If you are not sure if a list item applies to your research, read the appropriate section before selecting a response.

Materials & experimental systems

n/a	Involved in the study	
☒	☐	Antibodies
☒	☐	Eukaryotic cell lines
☒	☐	Palaeontology and archaeology
☒	☐	Animals and other organisms
☒	☐	Clinical data
☒	☐	Dual use research of concern
☒	☐	Plants

Methods

n/a	Involved in the study	
☒	☐	ChIP-seq
☒	☐	Flow cytometry
☐	☒	MRI-based neuroimaging

Plants

Seed stocks

Report on the source of all seed stocks or other plant material used. If applicable, state the seed stock centre and catalogue number. If plant specimens were collected from the field, describe the collection location, date and sampling procedures.

Novel plant genotypes

Describe the methods by which all novel plant genotypes were produced. This includes those generated by transgenic approaches, gene editing, chemical/radiation-based mutagenesis and hybridization. For transgenic lines, describe the transformation method, the number of independent lines analyzed and the generation upon which experiments were performed. For gene-edited lines, describe the editor used, the endogenous sequence targeted for editing, the targeting guide RNA sequence (if applicable) and how the editor was applied.

Authentication

Describe any authentication procedures for each seed stock used or novel genotype generated. Describe any experiments used to assess the effect of a mutation and, where applicable, how potential secondary effects (e.g. second site T-DNA insertions, mosiacism, off-target gene editing) were examined.

Magnetic resonance imaging

Experimental design

Design type

Magnetoencephalograpy: block design with one back task

Design specifications

8 blocks each with 10 trials, each trial shows an image for 2 s, and there is a 0.5 interval before the next trial.

Behavioral performance measures

button press, 95% correct criterion for each subject.

Acquisition

Imaging type(s)

Magnetoencephalography, with 3T structural MRI to map the MEG to the brain of each subject

Field strength

A MEGIN system was used for the MEG. A 3T Siemens Prisma was used for the structural.

Sequence & imaging parameters

MEG data were acquired on a TRIUX neo system at Zhangjiang Imaging Center (ZIC), containing 306 MEG sensors (102 magnetometers and 204 gradiometers). The sampling rate during data acquisition was 1000 Hz and an on-line band pass filter 0.03 to 330 Hz was applied. Prior to data recording, 5 Head Position Indicator (HPI) coils that were attached to the forehead of the subjects, as well as 3 anatomical fiducial points (two preauricular points and one nasion) were digitized using the FASTRAK Digitizer system for later co-registration with MRI data.
High-resolution structural T1-weighted MRI images were acquired in a 3T Siemens Prisma scanner at Zhangjiang Imaging Center (ZIC) with a 3D T1 MPRAGE sequence, field of view = 192 mm x 240 mm x 256 mm, 1 mm isotropic resolution, repetition time = 2.5 s, echo time = 2.15 ms, and flip angle = 8°.

Area of acquisition

Whole brain

Diffusion MRI

☒ Used ☐ Not used

Parameters

No data were collected. The data were from the Human Connectome project, as set out in the Methods.

Preprocessing

Preprocessing software

The MEG preprocessing was performed with the MNE-Python Version 1.5.1 software package (https://zenodo.org/records/8322569) . We used individual MRI images for head-modelling. The MRI data were pre-processed in Freesurfer V.7.1.4 (Fischel, 2012), and the head model (1-layer boundary element model, BEM, with the default conductivity of 0.3) was created in MNE-Python.

Normalization

All data were converted to HCP-MMP (Glasser et al 2016) with MNE-Python.

Normalization template

All data were converted to HCP-MMP (Glasser et al 2016) with MNE-Python.

Noise and artifact removal

First the environmental noise, recorded every day in the morning before testing, was suppressed from the raw MEG data with the spatio-temporal signal-space separation (SSP) method that is implemented in MNE-Python. A notch filter at 50 and 100 Hz was then applied, followed by a band-pass filter between 1 and 140 Hz. We further used the "find_bad_channels_maxwell" function from the MNE-Python software, plus visual inspection to identify bad channels, and further used the "maxwell_filter" function to implement bad channel reconstruction, movement compensation, and temporal Spatiotemporal Signal Space Separation (tSSS). On average, 5 channels were interpolated per participant. To suppress eye movement and cardio artefacts, we used the SSP method to remove 1 eye and 1 cardio component as provided by the MNE-Python software.

Volume censoring

N/A

Statistical modeling & inference

Model type and settings: A priori t-tests on MEG effective connectivities

Effect(s) tested: A priori t-tests on MEG effective connectivities to determine the direction of the effective conenctivity between groups of HCP-MMP regions.

Specify type of analysis: ☐ Whole brain ☒ ROI-based ☐ Both

Anatomical location(s): A-priori on cortical regions known to be involved in processing scenes from prior research.

Statistic type for inference (See Eklund et al. 2016): HCP-MMP cortical region based analyses: measurement of the magnitude and direction of the effective connectivity.

Correction: Only a few direction comparisons needed to be tested, and the criterion was set at p<0.01 to allow for the several tests.

Models & analysis

n/a	Involved in the study
☐	☒ Functional and/or effective connectivity
☒	☐ Graph analysis
☒	☐ Multivariate modeling or predictive analysis

Functional and/or effective connectivity: Effective connectivity algorithm of Deco and Rolls fully described in the paper and previously published.
